# Insights into the Complexation Mechanism of a Promising Lipophilic PyTri Ligand for Actinide Partitioning from Spent Nuclear Fuel

**DOI:** 10.1021/acs.inorgchem.2c02332

**Published:** 2022-11-04

**Authors:** Francesco Galluccio, Elena Macerata, Patrik Weßling, Christian Adam, Eros Mossini, Walter Panzeri, Mario Mariani, Andrea Mele, Andreas Geist, Petra J. Panak

**Affiliations:** † Department of Energy, Politecnico di Milano, Piazza Leonardo da Vinci 32, Milano20133, Italy; ‡ Karlsruhe Institute of Technology (KIT), Institute for Nuclear Waste Disposal (INE), P.O. Box 3640, Karlsruhe76021, Germany; § Institute for Physical Chemistry, Heidelberg University, Im Neuenheimer Feld 253, Heidelberg69120, Germany; ∥ C.N.R.—Consiglio Nazionale Delle Ricerche, Istituto di Scienze e Tecnologie Chimiche “G. Natta” (SCITEC), Sezione “U.O.S. Milano Politecnico”, Milan20133, Italy; ⊥ Department of Chemistry, Materials and Chemical Engineering “G. Natta”, Politecnico di Milano, Piazza Leonardo da Vinci 32, Milano20133, Italy

## Abstract

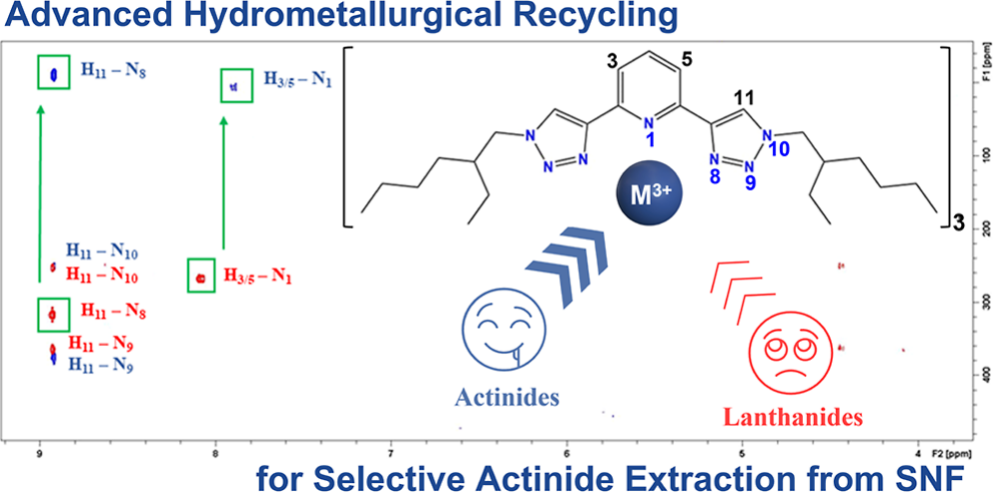

The challenging issue of spent nuclear fuel (SNF) management is being tackled by developing advanced technologies that point to reduce environmental footprint, long-term radiotoxicity, volumes and residual heat of the final waste, and to increase the proliferation resistance. The advanced recycling strategy provides several promising processes for a safer reprocessing of SNF. Advanced hydrometallurgical processes can extract minor actinides directly from Plutonium and Uranium Reduction Extraction raffinate by using selective hydrophilic and lipophilic ligands. This research is focused on a recently developed *N*-heterocyclic selective lipophilic ligand for actinides separation to be exploited in advanced Selective ActiNide EXtraction (SANEX)-like processes: 2,6-bis(1-(2-ethylhexyl)-1H-1,2,3-triazol-4-yl)pyridine (PyTri-Ethyl-Hexyl-PTEH). The formation and stability of metal–ligand complexes have been investigated by different techniques. Preliminary studies carried out by electrospray ionization mass spectrometry (ESI–MS) analysis enabled to qualitatively explore the PTEH complexes with La(III) and Eu(III) ions as representatives of lanthanides. Time-resolved laser fluorescence spectroscopy (TRLFS) experiments have been carried out to determine the ligand stability constants with Cm(III) and Eu(III) and to better investigate the ligand complexes involved in the extraction process. The contribution of a 1:3 M/L complex, barely identified by ESI–MS analyses, was confirmed as the dominant species by TRLFS experiments. To shed light on ligand selectivity toward actinides over lanthanides, NMR investigations have been performed on PTEH complexes with Lu(III) and Am(III) ions, thereby showing significant differences in chemical shifts of the coordinating nitrogen atoms providing proof of a different bond nature between actinides and lanthanides. These scientific achievements encourage consideration of this PyTri ligand for a potential large-scale implementation.

## Abstract 2

Over the last few years, the pyridine-bistriazole scaffold was found very selective toward minor actinides within advanced recycling strategies of SNF. In this research work, the complexation mechanism and the selectivity of a promising CHON lipophilic ligand belonging to the PyTri family, PTEH, has been investigated with lanthanides and actinides by ESI−MS, TRLFS, and NMR. The obtained results back up the recent selection of PTEH as an alternative ligand for the SANEX-like processes.

## Introduction

The reprocessing of spent nuclear fuel (SNF) and the recycling of plutonium and the minor actinides (MAs) (Np, Am, and Cm) into advanced nuclear fuels would increase the public acceptance of nuclear energy by improving the natural resources exploitation, reducing the long-term radiotoxicity and heat load of nuclear waste as well as repository constraints.^1−4^

Such a vision has boosted the development of several processes for the recovery of MAs from high-level waste, and a large number of hydrophilic and lipophilic extractants have been developed to achieve this challenging goal.^5−8^

In particular, the efforts have been focused on ligands bearing soft-donor atoms for their capability to interact more strongly with trivalent actinide ions rather than lanthanide ions. The need to control the generation of secondary waste leads to further restrict the interest to ligands fulfilling the CHON principle, that is, ligands composed of C, H, O, and N only in order to be completely incinerable at the end of their useful life.

In this perspective, the regular-Selective ActiNides EXtraction (*r*-SANEX) and 1cycle-SANEX (1c-SANEX) processes have been developed to separate trivalent MAs from the high active raffinate downstream of DIAMide EXtraction (DIAMEX) or Plutonium Uranium Reduction EXtraction (PUREX)-like processes using lipophilic heterocyclic aromatic *N*-donor bistriazinyl-pyridine (BTP), bistriazinyl-bipyridine (BTBP), and bistriazinyl-1,10-phenanthroline (BTPhen) ligands.^9−13^ To date, the European reference compound for An(III)/Ln(III) separation is the 6,6′-bis(5,5,8,8-tetramethyl-5,6,7,8-tetrahydro-benzo-1,2,4-triazin-3-yl)-2,2′-bipyridine, named CyMe_4_-BTBP.^14,15^

**Figure 1:**
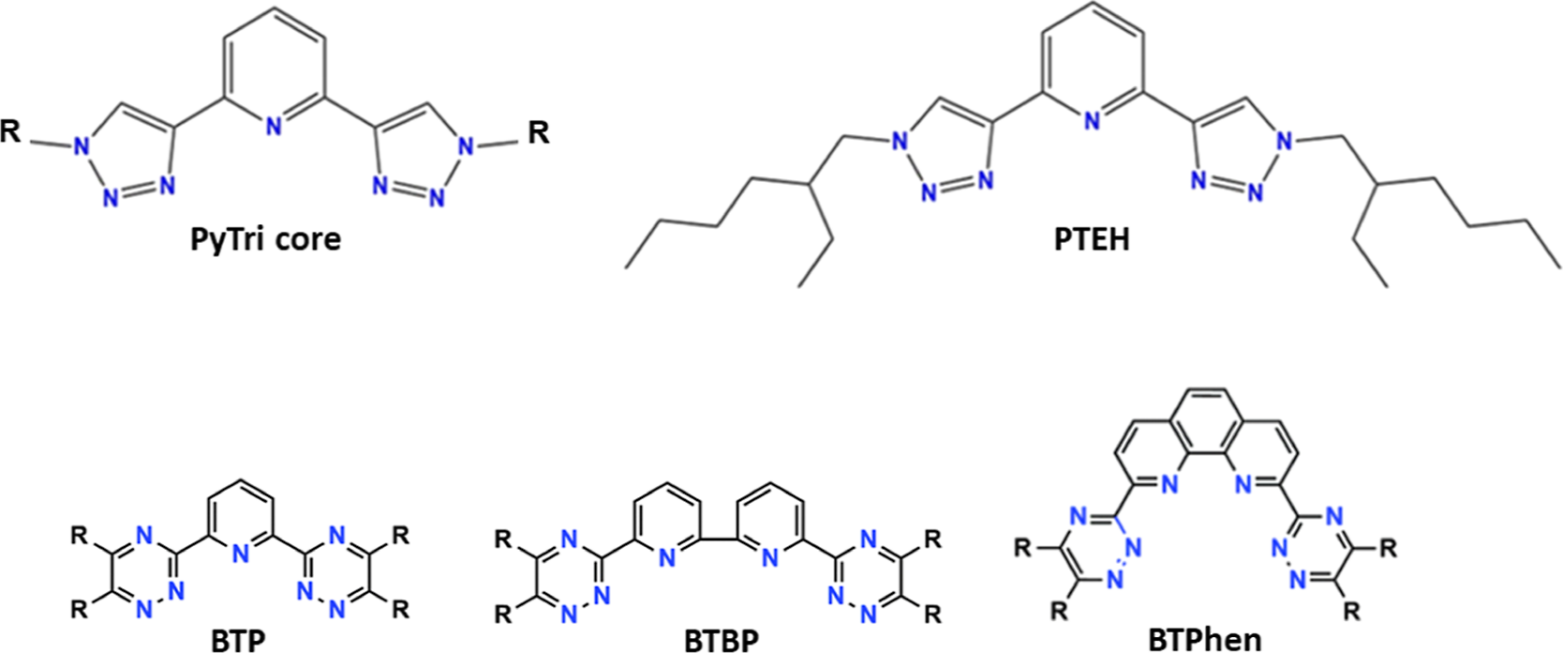
Top: molecular structures of the PyTri core (left) and of the lipophilic PTEH (right). Bottom: molecular structures of the heterocyclic aromatic *N*-donor BTP, BTBP, and BTPhen ligands.

Besides the widely investigated BTP, BTBP, and BTPhen ligands (Figure 1, bottom), recently, the pyridine-bistriazole (PyTri) chelating unit (Figure 1, top left) was found to be similarly promising for the selective An(III) separation under SANEX conditions.^16−18^ In particular, the lipophilic 2,6-bis(1-(2-ethylhexyl)-1H-1,2,3-triazol-4-yl)pyridine (PyTri-Ethyl-Hexyl—PTEH) ligand proved to be an excellent candidate for the SANEX-like processes (Figure 1, top right), endowed with a good solubility in the mixtures of organic diluents used, a satisfactory extraction efficiency, a remarkable An selectivity, a fast extraction kinetics and a good radiochemical stability.^19,20^

Given this promising experimental evidence and the prospective of an industrial application of such a PyTri family, the complexation behavior of the PTEH ligand has been investigated in details in the present work by means of different techniques. Indeed, fundamental studies on the complexation properties, together with the better understanding of the molecular reason for their remarkable selectivity, are paramount for the systematic improvement of such extracting agents toward the industrial application. Previous results suggested that cation extraction in the presence of PTEH is achieved by a mixture of 1:2 and 1:3 metal/ligand complex stoichiometries, with the prevailing 1:2 stoichiometry.^18^

Preliminary studies were performed by electrospray ionization mass spectrometry (ESI–MS) to qualitatively investigate the PTEH complexes with stable La(III) and Eu(III) metal cations and attempting to shed light on the complexation mechanism involved in the extraction process.^21,22^

Time-resolved laser fluorescence spectroscopy (TRLFS) was used to investigate the formation of different Cm(III) and Eu(III) complex species in sub-micro molar concentrations and to determine the conditional stability constants. Finally, investigations on PTEH complexes with Lu(III) and Am(III) were conducted by NMR spectroscopy to deepen the understanding of the different types of metal–ligand bonding, which could play an important role for the selectivity of some *N*-donor ligands in the complexation process.^23,24^

## Experimental Section

### Chemicals

2,6-bis(1-(2-ethylhexyl)-1H-1,2,3-triazol-4-yl)pyridine (PTEH) was supplied by University of Parma (Department of Chemistry, Life Sciences and Environmental Sustainability) and synthetized according to the procedure elsewhere reported.^11^

All commercially available reagents and chemicals used in this study were of analytical reagent grade and used without further purification. Kerosene (low odor, aliphatic fraction >95%) and 1-octanol (purity ≥99%), both from Sigma-Aldrich company, were used as diluents.

Nitric acid solutions were prepared by diluting concentrated nitric acid (from FLUKA, ≥65% w/w) with deionized water. Hexahydrated nitrates of La and Eu (purity from 99 to 99.99%), purchased from Sigma-Aldrich, were used to prepare simplified synthetic feed stock solutions in HNO_3_ at different concentrations.

Methanol was of spectroscopy grade (Uvasol Supelco from Merck). Deuterated solvents were purchased from Euriso-Top GmbH. Am(OTf)_3_ was prepared at INE-KIT Research Centre, while Lu(OTf)_3_ was purchased from Sigma-Aldrich.

### ESI–MS Sample Preparation

#### Monophasic Solutions

The ligand stock solution was prepared by dissolving 87.5 mg of PTEH in 1 mL of a kerosene/1-octanol mixture with 10 vol % 1-octanol content to ensure good ligand solubility and to prevent third phase formation during the extraction process, obtaining a 0.2 M stock solution. One more solution, containing 10^–4^ M PTEH, was prepared by successive dilution of the stock solution in acetonitrile. The La(III) stock solution was prepared by dissolving 86.6 mg of La(NO_3_)_3_· 6 H_2_O in 1 mL of 3 M HNO_3_ to obtain a 0.2 M solution, then diluted to 10^–4^ M. The solutions of Eu(III) nitrate were prepared in the same way as those of La(III) nitrate. Monophasic solutions containing the ligands (L) and the metal cations (M) were prepared by mixing proper volumes of suitable stock solutions described above, in order to adjust the desired [L]/[M] ratios, and subsequently diluted in acetonitrile to 10^–4^ M.

#### Extraction Samples

The organic phase consisted of a mixture of kerosene with 10 vol % 1-octanol. An amount of 87 mg of PTEH was dissolved in 1 mL of the organic mixture to obtain a 0.2 M ligand solution. Concerning the aqueous phase, 86 mg of La(NO_3_)_3_· 6 H_2_O was dissolved in 3 M HNO_3_ to properly simulate the acidic medium of the extraction process. Afterward, 300 μL of both phases was mixed using a shaker at controlled temperature of 22 °C and at velocity of 2000 rpm for 1 h. Following centrifugation at 6000 rpm for 10 min, 200 μL of organic and aqueous phases was transferred into two vials, then diluted to 10^–4^ M before measuring.

### ESI–MS Measurements

ESI–MS experiments were carried out using a Bruker Esquire 3000 PLUS instrument (ESI Ion Trap LC/MS^n^ System), equipped with an ESI source and a quadrupole ion trap detector. Sample infusion in the ESI–MS was performed at 4 μL/min rate. The analyses were performed in positive and negative ion modes after optimization of instrument conditions: 4.5 kV needle voltage, 10 L/h N_2_ flow rate, 40 V cone voltage, and 100–1200 mass/charge range. The assignment of peaks was supported by collision induced dissociation (CID) tandem mass spectrometry experiments, and by comparison of the experimental isotopic pattern with the calculated one. As the ion trap detector (ITD) allows for multiple collisions, the general notation of MS^*n*^ is also used in the manuscript as synonym of multiple CID mass spectra.

### TRLFS Sample Preparation

#### Titrations

8.7 mg of PTEH was dissolved in 200 μL of methanol with 5 vol % water content to obtain a 0.1 M stock solution. Three more solutions, containing 10^–2^ M, 10^–3^ M, and 10^–4^ M PTEH, were prepared by successive dilutions. The Cm(III) sample was prepared by adding 4.72 μL of a 2.12 × 10^–5^ M Cm(ClO_4_)_3_ stock solution (0.1 M HClO_4_; ^248^Cm: 89.7%, ^246^Cm: 9.4%, ^243^Cm: 0.4%, ^244^Cm: 0.3%, ^245^Cm: 0.1%, and ^247^Cm: 0.1%), 5 μL of 2 M HClO_4_, 40.28 μL of water, and 950 μL of methanol, resulting in an initial Cm(III) concentration of 1 × 10^–7^ M and a proton concentration of 0.01 M. The Eu(III) sample was prepared by adding 9.34 μL of a 1.07 × 10^–3^ M Eu(ClO_4_)_3_ stock solution in 0.1 M HClO_4_, 5 μL of 2 M HClO_4_, 35.66 μL of water, and 950 μL of methanol, resulting in an initial Eu(III) concentration of 1 × 10^–5^ M and a proton concentration of 0.01 M.

#### Extraction Tests

The organic phase for the extraction process consisted of a kerosene/1-octanol mixture with 10 vol % 1-octanol. An amount of 35 mg of PTEH was dissolved in 400 μL of the organic phase to obtain a 0.2 M solution. The aqueous phase consisted of 400 μL of spiked solution with 10^–7^ M Cm(III) in 3 M HNO_3_ for the Cm(III) extraction test and 400 μL of spiked solution with 10^–5^ M Eu(III) in 3 M HNO_3_ for the Eu(III) experiments. The aqueous and organic phases were mixed on an orbital shaker at controlled temperature of 20 °C and at velocity of 2000 rpm for 1 h. Following centrifugation at 6000 rpm for 10 min, 300 μL of both phases was separated and transferred into quartz cuvettes for TRLFS analysis without dilution.

### TRLFS Measurements

TRLFS measurements were performed at 298 K using a Nd/YAG (Surelite II laser, Continuum) pumped dye laser system (NarrowScan D-R; Radiant Dyes Laser Accessories GmbH). The wavelengths of 396.6 nm and 394 nm were used to excite Cm(III) and Eu(III) ions, respectively. A spectrograph (Shamrock 303i, ANDOR) with 300, 1199, and 2400 lines per mm gratings was used for spectral decomposition. The fluorescence emission was detected using an ICCD camera (iStar Gen III, ANDOR) after a delay time of 1 μs to discriminate short-lived organic fluorescence and light scattering.

### NMR Sample Preparation

The ligand solution was prepared by dissolving 7.87 mg (18 μmol) of PTEH in 600 μL of pure deuterated methanol (CD_3_OD) with traces of tetramethylsilane (TMS), thus obtaining a 0.03 M PTEH solution. The Lu-PTEH solution was prepared in pure deuterated methanol (CD_3_OD) by adding a stoichiometric amount (6 μmol) of Lu(III) triflate salt to the PTEH solution (Lu/PTEH molar ratio equal to 1:3). After mixing, the [Lu(PTEH)_3_](OTf)_3_ complex solution was transferred into an NMR tube for measuring. The Am-PTEH solution was prepared by evaporating 6 μmol of Am(OTf)_3_ solution. The residue was then dissolved in the ligand solution (Am/PTEH molar ratio equal to 1:3). Afterward, the solution was carefully mixed and transferred into a J. Young-type NMR tube for measuring.

### NMR Measurements

NMR spectra were recorded at *T* = 300 K on a Bruker Avance III 400 spectrometer operating at a resonance frequency of 400.18 MHz for ^1^H nuclei. The spectrometer was equipped with a z-gradient observe room temperature probe. Chemical shifts were referenced internally to tetramethylsilane (TMS) (δ(TMS) = 0 ppm) by the deuterium lock signal of D_2_O. For single-scan ^1^H spectra, standard 90° pulse sequences were used. All spectra were recorded with 32 k data points and were zero filled to 64 k points.

## Results and Discussion

### ESI–MS: Investigations of La(III) and Eu(III) Speciation with PTEH

Preliminary spectra were recorded in order to check the pure components of the system under study. As shown in Figure S1, the most intense peaks in the pure PTEH spectrum are at *m/z* 438.3, 460.3, 476.3, and 897.7, assigned, in the order, to protonated PTEH, to sodium cationized [PTEHNa]^+^, potassium cationized [PTEHK]^+^, and to the dimeric sodium adduct [(PTEH)_2_Na]^+^, as reported in Table S1. Besides, the pure La(III) nitrate spectrum was recorded in the negative ion mode (−MS) as a control aqueous phase for data comparison after the extraction tests with PTEH. The major detectable and assigned signals, as noticeable in Figure S2 and Table S2, are at *m/z* 386.5, 711.6, and 796.6, corresponding to [La(NO_3_)_4_]^-^, [La_2_(NO_3_)_7_]^-^, and [La_2_Na(NO_3_)_8_]^-^, respectively. Peak assignment was supported by tandem mass spectrometry and by simulated isotopic patterns (Supporting Information).

#### La(III) Speciation with PTEH

ESI–MS spectra of La(III) nitrate with PTEH (L) in monophasic solutions containing 3 M HNO_3_ were recorded at different [L]/[M] ratios, namely, 1, 5, and 10. Monophasic solutions without nitric acid were analyzed at the same [L]/[M] ratios for comparison to highlight the influence of the acidity on the complex formation.

**Figure 2:**
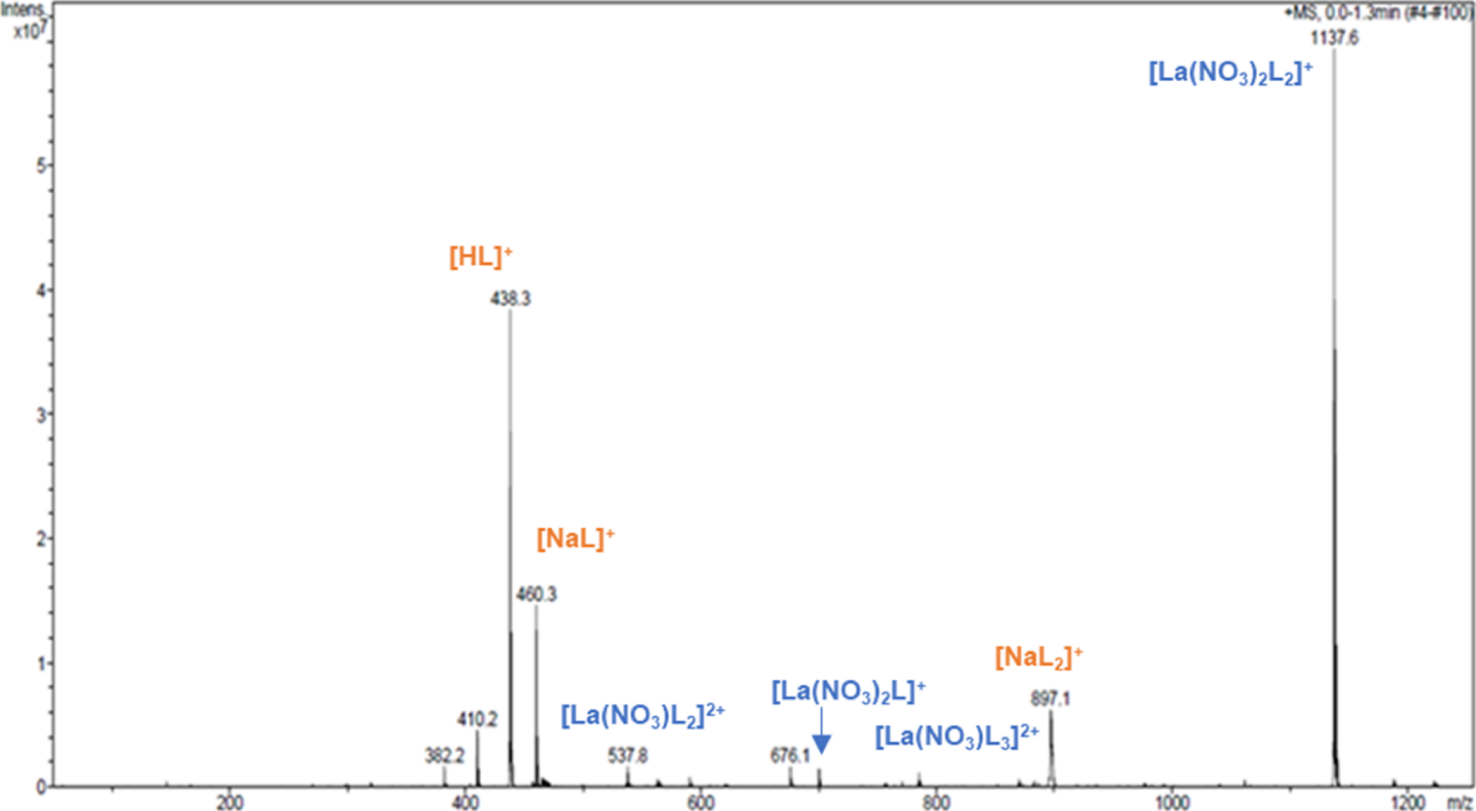
Positive ESI–MS spectrum of the [L]/[M] = 1 solution containing La(III) nitrate in 3 M HNO_3_ and PTEH in kerosene with 10 vol % 1-octanol, diluted in acetonitrile.

**Figure 3:**
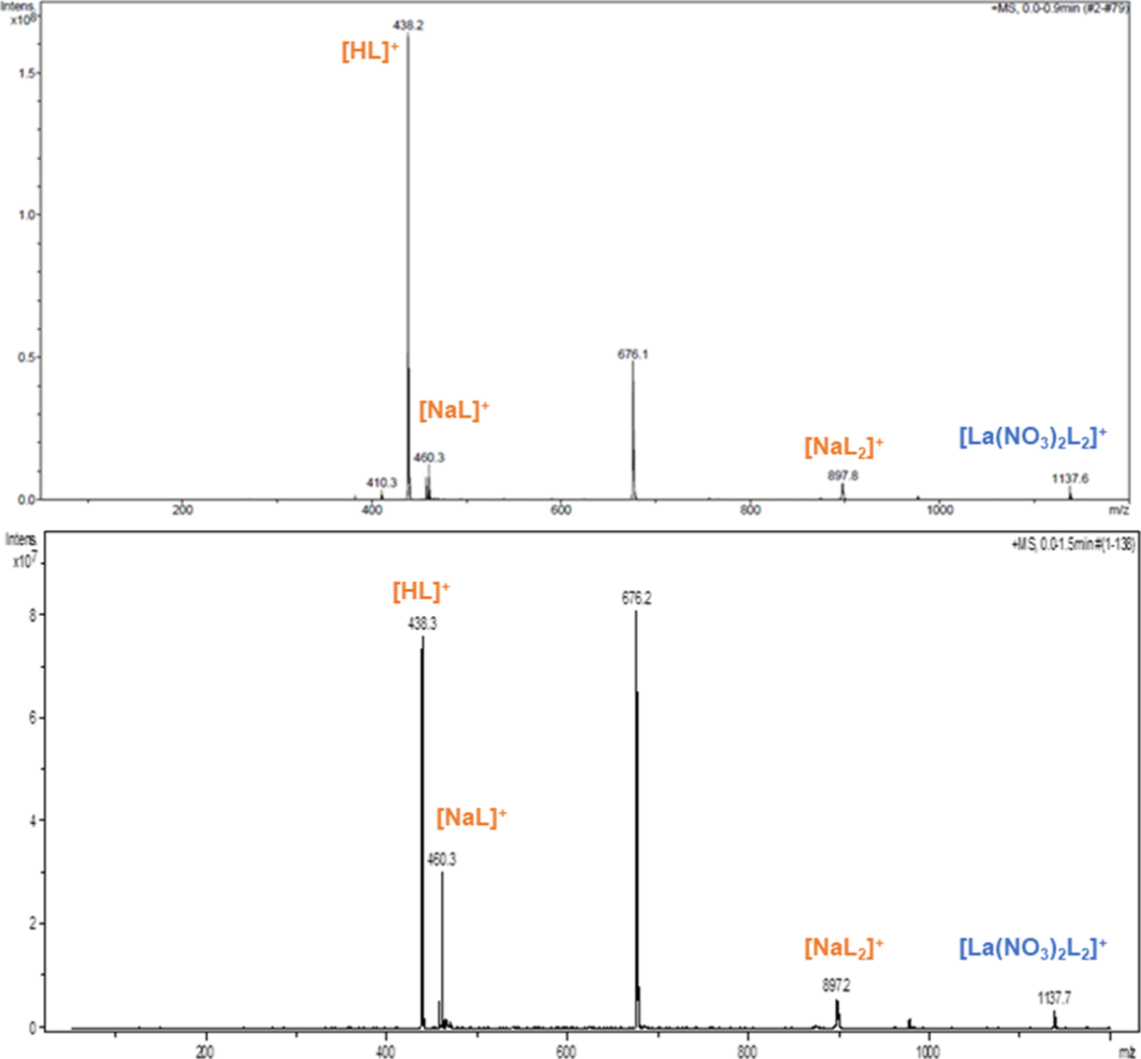
Positive ESI–MS spectra of solutions containing La(III) nitrate in 3 M HNO_3_ and PTEH in kerosene with 10 vol % 1-octanol at a [L]/[M] ratio equal to 5 (top) and 10 (bottom), diluted in acetonitrile.

The spectrum of the [L]/[M] = 1 solution is displayed in Figure 2, where the major complex species with La(III) appear along with the ligand adducts. The complex stoichiometry and the species designation are reported in Table 1. The base peak at *m/z* 1137.5 was assigned to the complex of formula [La(NO_3_)_2_L_2_]^+^ showing the presence of two nitrate ions in the coordination sphere and two complexing ligands. Peak assignment was further confirmed by tandem mass spectrometry (Supporting Information). In Figure S3, the MS^2^ spectrum of the parent peak at *m/z* 1137.5 is reported. The major fragment appears at *m/z* 700.4, consistent with [La(NO_3_)_2_L]^+^ originated by the loss of one ligand molecule. As can be seen in Figure S4, the MS^2^ spectrum of the parent ion at *m/z* 700.4 shares many fragment peaks with the fragmentation of *m/z* 1137.6, thus confirming the assignment and the stoichiometry of the major complex species. The first fragmentation pathway shows a neutral loss of 112 mass units, along with some peaks differing in 28 mass units, presumably C_2_H_4_ fragment from the ligand. The predicted 1:3 La(III) complex with PTEH at *m/z* 756.4 was barely observed in the spectrum of the equimolar solution (Figure 2). Notably, a small peak at *m/z* 757 was isolated and attributed to the 1:3 [La(NO_3_)L_3_]^2+^ complex by the simulated isotopic pattern and its MS^2^ spectrum (Figure S5), that shows the 1:2 [La(NO_3_)L_2_]^2+^ complex thereby confirming the previous peak assignments. Some experiments were performed by changing the [L]/[M] ratio from 1 to 10. The speciation spectrum does not show any remarkable variation, see Figure 3. Conversely, a decreasing trend in stability of the lower complex stoichiometry can be observed. In particular, the peaks at *m/z* 537.8, 700.4, and 756.4 are not easily visible anymore in the spectrum. Besides, the relative intensity of the major 1:2 complex at *m/z* 1137.5 is lower than before. A large increase in intensity is conversely observed for the unidentified species at *m/z* 676.2.

**Table 1:** Species Designation and Stoichiometry of the [L]/[M] = 1 Solution Containing La(III) Nitrate in 3 M HNO_3_ and PTEH in Kerosene With 10 Vol % 1-Octanol, Diluted in Acetonitrile

*M*/*L*	species	*m/z*
1:2	[La(NO_3_)L_2_]^2+^	537.8
1:1	[La(NO_3_)_2_L]^+^	700.4
1:3	[La(NO_3_)L_3_]^2+^	756.4
1:2	[La(NO_3_)_2_L_2_]^+^	1137.5

The influence of the acidic medium on the complexation was also investigated by repeating the experiments on solutions with the same [L]/[M] ratio, containing La(III) nitrate in 3 M HNO_3_ and PTEH in kerosene with 10 vol % 1-octanol. The spectrum of the equimolar solution is reported in Figure S6, showing that also in this case, the [La(NO_3_)_2_L_2_]^+^ species gives rise to an intense signal at *m/z* 1137.5. Accordingly, the 1:2 complex stoichiometry seems to be the most easily detectable, thus revealing a good stability in the gas phase despite the absence of nitric acid in the injected solution. By increasing [L]/[M] ratios, the complex at *m/z* 700.4 assigned to [La(NO_3_)_2_L]^+^ shows non-negligible intensity, thus indicating considerable stability of such a La(III) complex at these experimental conditions (Figure S7). The complex stoichiometry at a [L]/[M] ratio equal to 10 was also explored to find any 1:3 La(III) species like [La(NO_3_)L_3_]^2+^ complex at *m/z* 756.4 favored by the increased ligand concentration (Figure S8). Conversely, the 1:2 stoichiometries, despite their intensity, reveal weaker complexation stability than before. All the results obtained so far are consistent with those coming from the monophasic solutions containing 3 M HNO_3_, thereby revealing the negligible influence of the acidity on the formed species.

#### Eu(III) Speciation with PTEH

Speciation spectra of equimolar solutions containing Eu(III) nitrate and PTEH in acetonitrile with 3 M HNO_3_ were recorded in the positive ion mode with target mass of *m/z* 500 and 1500 (Supporting Information). As in the speciation studies with La(III) nitrate, the positive mono charged [Eu(NO_3_)_2_L_2_]^+^ species is the most intense signal in the spectrum; other relevant complex species are observed at *m/z* 544.8, 714.3, and 763.4. In addition, minor species can be observed at *m/z* 488.3 and 513.8, which seem to come from Eu(II) complexes. Indeed, complexes with Eu(II) and Eu(III) can be expected according to the literature.^25^ Complex stoichiometry and species identification are reported in Table 2.

**Table 2:** Species Designation and Stoichiometry of the Equimolar Solution Containing Eu(III) Nitrate and PTEH in Acetonitrile with Nitric Acid

*M*/*L*	species	*m/z*
1:2	[EuL_2_]^2+^	513.8
1:2	[Eu(NO_3_)L_2_]^2+^	544.8
1:1	[Eu(NO_3_)_2_L]^+^	714.2
1:3	[Eu(NO_3_)L_3_]^2+^	763.4
1:2	[Eu(NO_3_)_2_L_2_]^+^	1151.6

**Figure 4:**
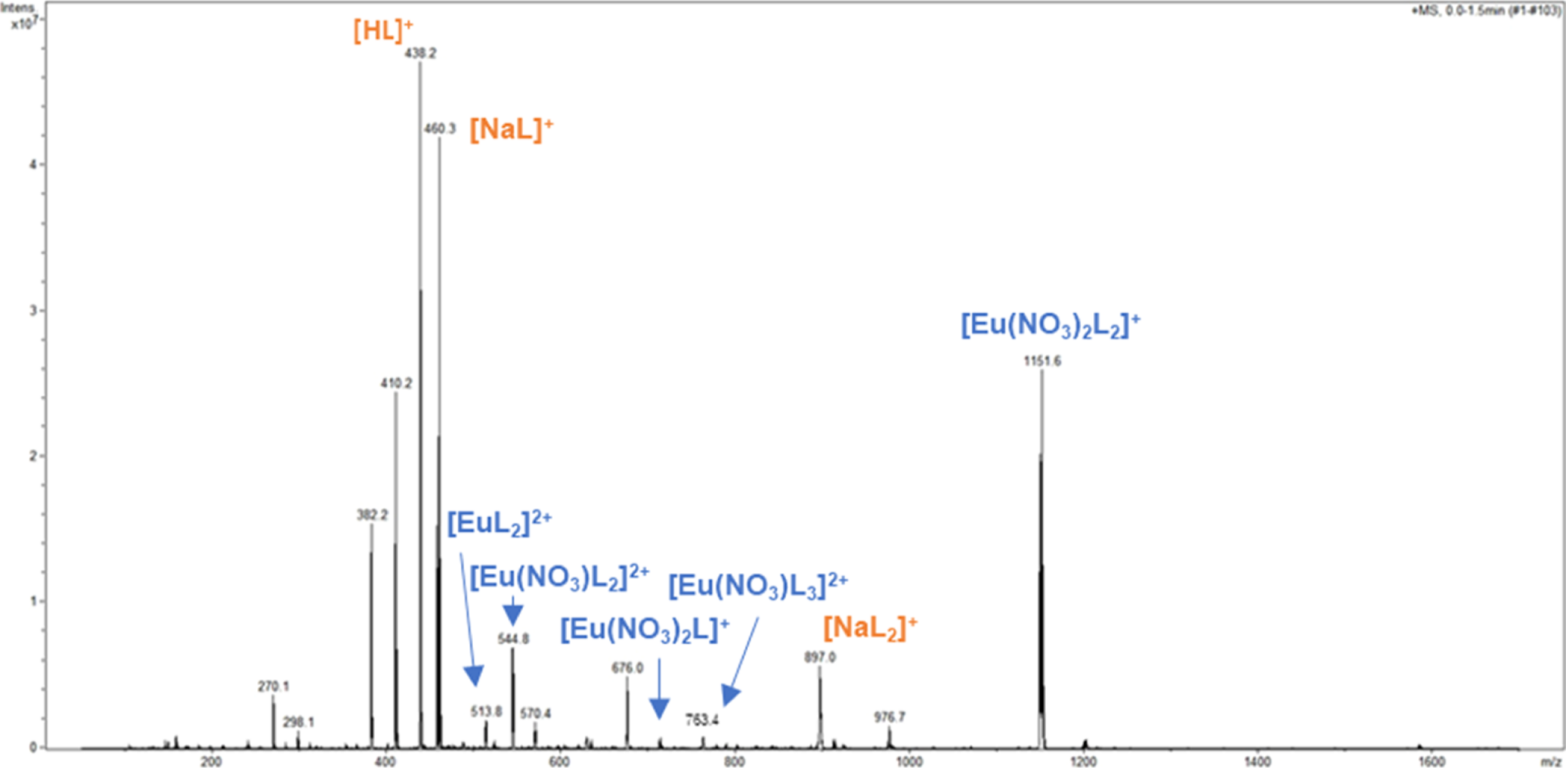
Positive ESI–MS spectrum of the solutions containing Eu(III) nitrate and PTEH at a [L]/[M] ratio equal to 5 with 3 M HNO_3_.

Afterward, multiple tandem mass spectrometry was performed to confirm the species designation. According to the complexation mechanism, an increase in the ligand concentration results in higher complex stoichiometries. At a [L]/[M] ratio equal to 5, the major complex species are clearly confirmed by the speciation spectrum recorded in positive high mass and displayed in Figure 4: a small increase in the relative intensity of the 1:2 [Eu(NO_3_)L_2_]^2+^ complex species at *m/z* 544.8 is observed in the spectrum. MS^n^ fragmentation analysis was performed for some relevant peaks, thereby also confirming the identified complex species. The speciation spectrum at a [L]/[M] ratio equal to 10 showed that the 1:2 [Eu(NO_3_)_2_L_2_]^+^ complex now appears at *m/z* 1149.6, but its assignment was confirmed by comparing the experimental spectrum with the simulated isotopic pattern (Supporting Information). The dominant 1:2 [Eu(NO_3_)_2_L_2_]^+^ complex is less intense, whereas the higher stoichiometries such as the 1:3 [Eu(NO_3_)L_3_]^2+^ species seems to be favored and it appears more intense than before. The unidentified species at *m/z* 676.5 was also found in the europium experiments. Although a peak assignment was attempted by mass tandem spectrometry (Figure S9), the species remains unknown.

#### La(III) Extraction Tests

**Figure 5:**
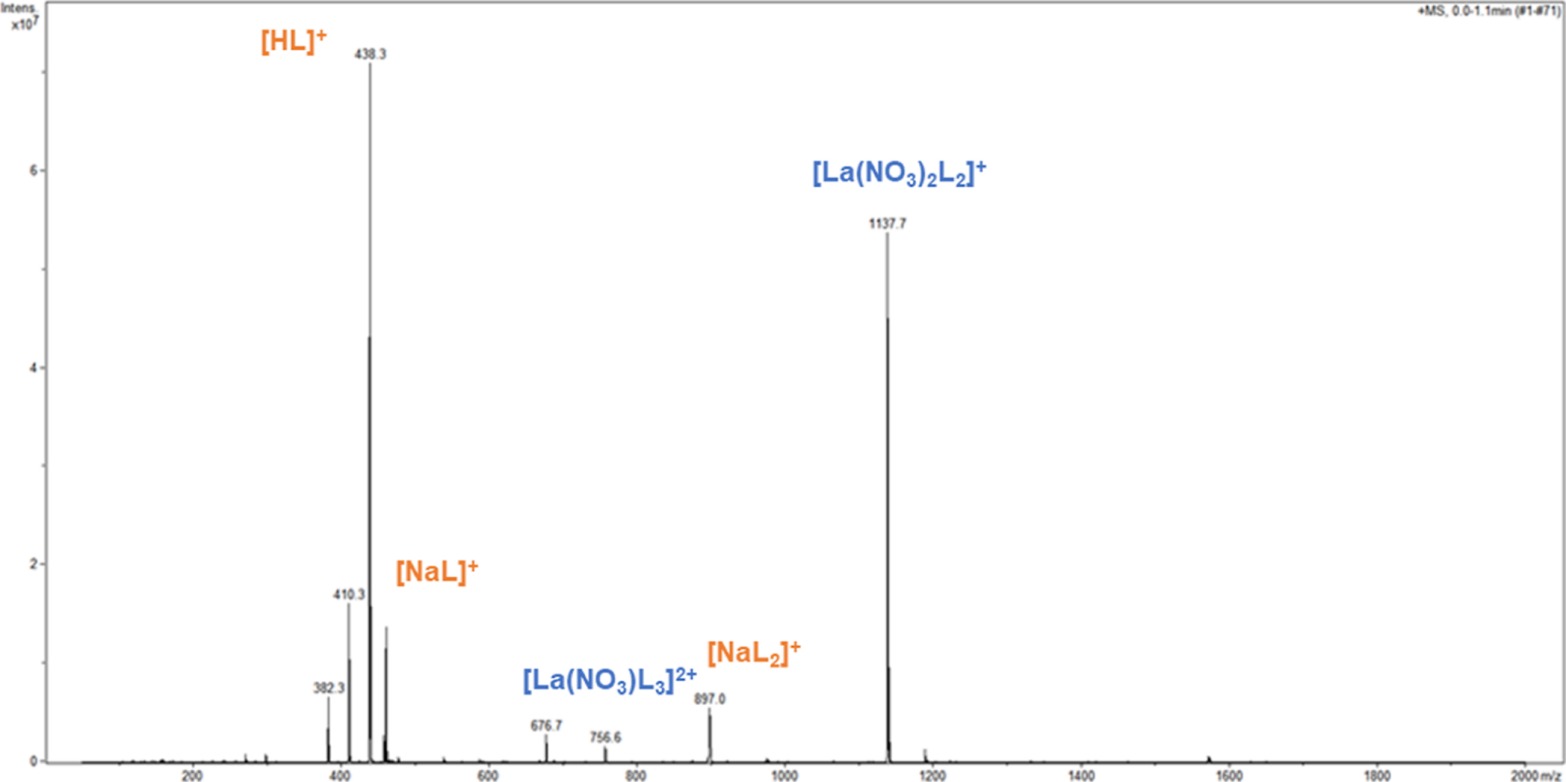
ESI–MS spectrum of the organic phase after the extraction of La(III) in 3 M HNO_3_ into 0.2 M PTEH dissolved in a kerosene with 10 vol % 1-octanol mixture.

The spectrum of the loaded organic phase shows all the major species formed following the extraction process, thus providing information about the La(III) speciation with PTEH at a [L]/[M] ratio equal to 1. Figure 5 shows the [La(NO_3_)_2_L_2_]^+^ complex as dominant in the extraction process under these experimental conditions and in agreement with the results coming from the monophasic solutions at the same [L]/[M] ratio. In addition, a small contribution of the 1:3 [La(NO_3_)L_3_]^2+^ complex at *m/z* 756.4 is evident in the extraction process.

To sum up, the PTEH speciation with Eu(III) nitrate at an increasing ligand concentration is coherent with monophasic La(III) experiments, even though in this case, the 1:3 complex appears more intense and almost as dominant as the 1:2 [Eu(NO_3_)_2_L_2_]^+^ complex species.

### TRLFS: Complexation of Cm(III) and Eu(III) with PTEH

#### Titrations

**Figure 6:**
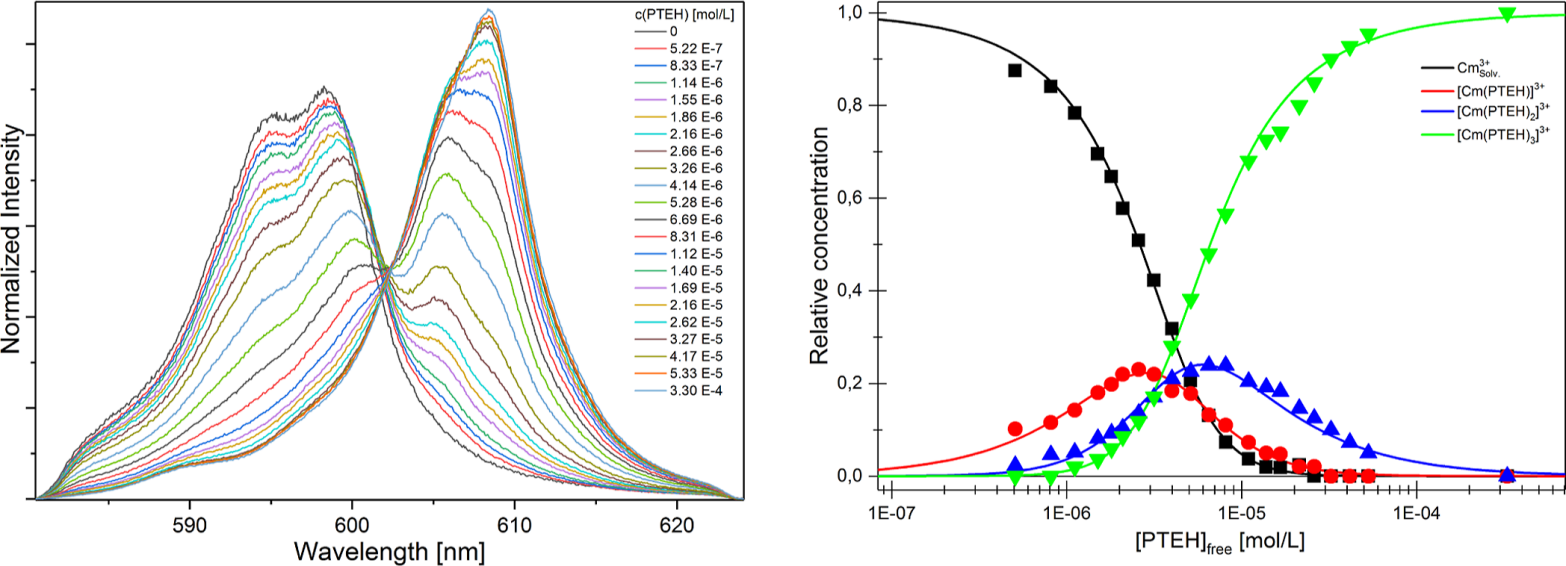
Left: Evolution of the normalized fluorescence spectra of Cm(III) at increasing PTEH concentration in methanol with 5 vol % water ([Cm(III)]_ini_ = 1 × 10^–7^ M, [PTEH] = (0–3.30) × 10^–4^ M, [H]^+^ = 0.01 M). Right: Cm(III) species distribution as a function of the free PTEH concentration in methanol with 5 vol % water. Symbols denote the experimental data, while lines are calculated using log β_1:1_ = 5.2, log β_1:2_ = 10.7, and log β_1:3_ = 16.2 (*T* = 20 °C).

The Cm(III) fluorescence evolution resulting from the ^6^D′_7/2_ → ^8^S′_7/2_ transition was followed as a function of the ligand concentration, as depicted in Figure 6 (left). At zero ligand concentrations, the solvated Cm(III) ion has two emission bands at 594.0 nm and 599.0 nm. The spectrum shows a bathochromic shift with respect to the Cm(III) aquo ion [Cm(H_2_O)_9_]^3+^ located at 593.8 nm. This shift is due to a partial replacement of water molecules in the inner Cm(III) ion coordination sphere by methanol molecules. At increasing PTEH concentration, three new emission bands located at 600.3, 605.8, and 608.4 nm grow up step by step. Again a bathochromic shift can be observed with respect to the solvated Cm(III) ion, due to the increased splitting of the ^6^D′_7/2_ state upon complexation with PTEH. The new emission bands are attributed to the formation of new complex species: [Cm(PTEH)_n_]^3+^ (*n* = 1–3). The Cm(III) fluorescence spectrum evolved continuously with increasing ligand concentrations up to 2.62 × 10^–5^ M, that is, the last titration steps did not change the spectrum anymore.

The Cm(III) species distribution for each titration step was determined by peak deconvolution of the fluorescence spectra using the pure component spectra (Supporting Information) using Origin (for a more detailed description of peak deconvolution, see references).^26,27^ As shown in Figure 6 (right), at a low ligand concentration, [Cm_Solv._]^3+^ is the dominant species. At increasing PTEH concentration, the [Cm(PTEH)]^3+^ complex is formed with a maximum fraction of about 25% at 2.5 × 10^–6^ M of PTEH. Upon further addition of the ligand, the [Cm(PTEH)_2_]^3+^ complex is present with a relative fraction of 25% at 6 × 10^–6^ M PTEH. At higher ligand concentrations, the [Cm(PTEH)_3_]^3+^ complex is the dominant species in the solution.

The stoichiometry of the [Cm(PTEH)_n_]^3+^ (*n* = 1–3) complexes was evaluated by slope analyses. The formation of the complex species can be described by the following complexation reaction (1) (L = PTEH).
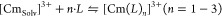
1

The stability constants log β′ for the Cm(III)-PTEH complexes were calculated according to eq 2. They are given in Table 3.
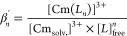
2

**Table 3:** Logarithmic Conditional Stability Constants Log β′_*n*_ of the [Cm(PTEH)_n_]^3+^ and [Eu(PTEH)_*n*_]^3+^ (*n* = 1–3) Complexes, *T* = 20 °C

ion/stability constants	logβ′_1:1_	logβ′_1:2_	logβ′_1:3_
Cm(III)	5.2 ± 0.1	10.7 ± 0.1	16.2 ± 0.3
Eu(III)	4.8 ± 0.1		14.1 ± 0.3

From eq 2, a linear correlation between the logarithm of the concentration ratio [Cm(PTEH)_*n*_]^3+^/[Cm_Solv._]^3+^ (*n* = 1–3) and the logarithm of the free PTEH concentration with a slope of *n* was drawn, as shown by eq 3.
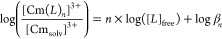
3

A double logarithmic plot showing the concentration ratios of the complexed species and the solvated metal ion related to the free ligand concentration is displayed in Figure S16. Linear regression analysis yields slopes of 0*.*96 ± 0*.*03, 2*.*00 ± 0*.*04, and 2*.*92 ± 0*.*03 for the formation of the [Cm(PTEH)_n_]^3+^ complexes (*n* = 1–3), verifying the postulated complexation model and confirming the accurate deconvolution as well as the correct assignment of the Cm(III)-PTEH complexes with emission maxima at 600*.*3, 605*.*8, and 608*.*4 nm.

**Figure 7:**
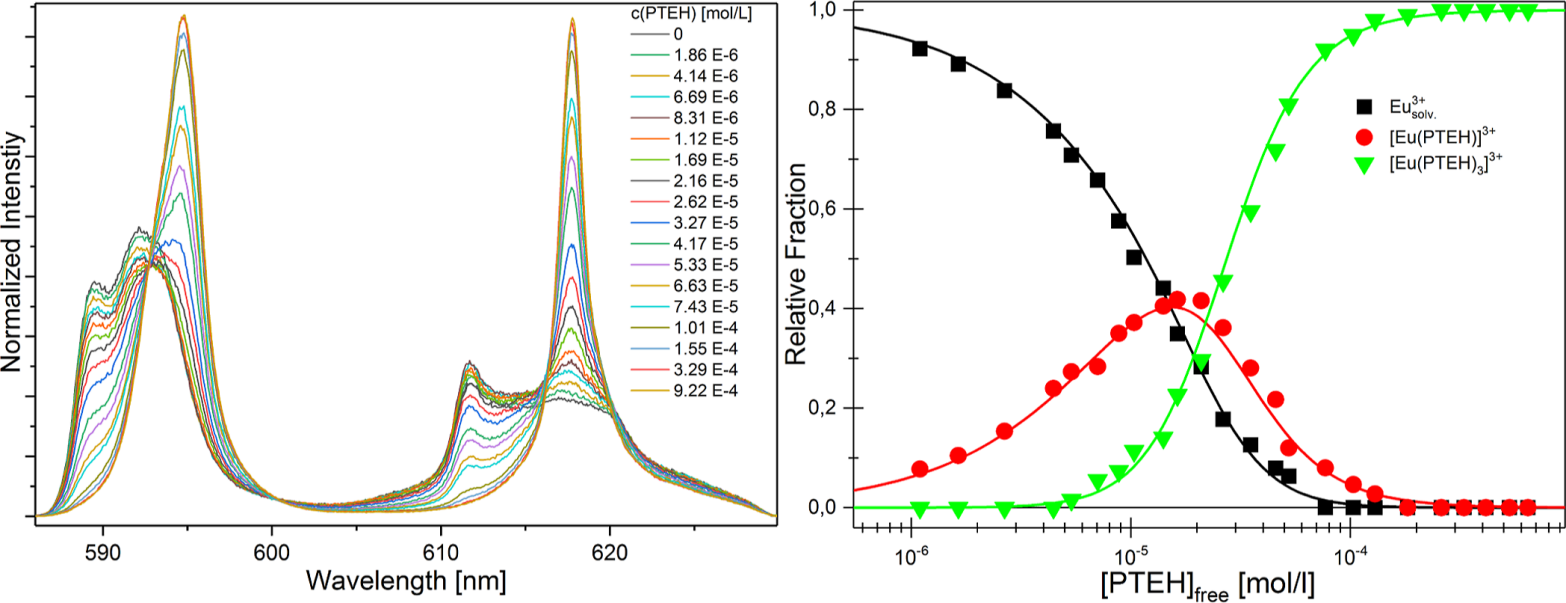
Left: Normalized fluorescence spectra of Eu(III) (^5^D_0_ → ^7^F_1_ and ^5^D_0_ → ^7^F_2_) at increasing ligand concentrations in methanol with 5 vol % water ([Eu(III)]_ini_ = 1 × 10^–5^ M, [PTEH] = (0–1.10) × 10^–2^ M, [H]^+^ = 0.01 M). Right*:* Eu(III) species distribution as a function of the PTEH free concentration in methanol with 5 vol % water. Symbols denote the experimental data, while lines are calculated using log β_1:1_ = 4.8 and log β_1:3_ = 14.1. *T* = 20 °C.

In the case of Eu(III), the evolution of the ^5^D_0_ → ^7^F_1_ and ^5^D_0_ → ^7^F_2_ emission bands was followed as a function of the PTEH concentration in methanol with 5 vol % water (as shown in Figure 7). The fluorescence spectrum of the solvated Eu(III) species shows two emission bands with maxima at 589*.*5 and 592*.*3 nm for the ^5^D_0_ → ^7^F_1_ transition and maxima at 611*.*7 nm and 617*.*4 nm for the ^5^D_0_ → ^7^F_2_ transition. At increasing PTEH concentration, the emission intensity of the solvent species decreases in favor of spectra of new complex species. The complexation of Eu(III) with PTEH leads to a splitting of the ^5^D_0_ → ^7^F_1_ and ^5^D_0_ → ^7^F_2_ emission bands. The shape of the ^5^D_0_ → ^7^F_1_ emission band changes toward a single peak with maximum at 594*.*8 nm, whereas the ^5^D_0_ → ^7^F_2_ emission band changes toward a peak with maximum at 617*.*8 nm. Changes of the Eu(III) emission spectra start at 1*.*86 × 10^–6^ M ligand concentration, and they are attributed to the formation of new [Eu(PTEH)_n_]^3+^ (*n* = 1, 3) complexes. No further changes are observed above 3*.*29 × 10^–4^ M PTEH concentration. Concerning the ^5^D_0_ → ^7^F_1_ and ^5^D_0_ → ^7^F_2_ transitions, the species distribution for each titration step was determined by peak deconvolution of the fluorescence spectra (Figure 7), using the pure component spectra, as shown in Figure S17. As shown in Figure 7, at low ligand concentrations, [Eu_Solv._]^3+^ is the dominant species. Upon further addition of ligands, the [Eu(PTEH)]^3+^ complex is formed with a maximum fraction of about 40% at 1*.*5 × 10^–5^ M of PTEH. With increasing PTEH concentrations, the [Eu(PTEH)_3_]^3+^ complex forms and becomes the dominant species in the solution at higher ligand concentrations. The complexation reaction from 1:1 to 1:3 species seems to be favored, that is, the [Eu(PTEH)_2_]^3+^ is present in such a small amounts that its contribution can be considered negligible in the investigated system. Stability constants of the Eu(III) complexes were calculated according to eq 2. They are given in Table 3. The double logarithmic plot of the concentration ratio of the different species in solution at various free ligand concentrations is reported in the Supporting Information. The linear regression analyses according to eq 3 yield slopes of 0*.*98 ± 0*.*02 and 2*.*90 ± 0*.*21 for the formation of the [Eu(PTEH)_n_]^3+^ (*n* = 1 and 3) complexes. The obtained results are in good agreement with the postulated complexation model and confirm the correct assignment of the Eu(III)-PTEH complexes.

The average values of the logarithmic conditional stability constants for the [Cm(PTEH)_n_]^3+^ and [Eu(PTEH)_n_]^3+^ complexes are reported in Table 3. PTEH forms more stable complexes with Cm(III) than that with Eu(III). This difference becomes more pronounced for higher complexed species. Following these experimental activities, a difference of 2 orders of magnitude between the stability constants of the [Cm(PTEH)_3_]^3+^ and [Eu(PTEH)_3_]^3+^ complex species was observed. This yields a theoretical separation factor  = 126 ± 4, in good agreement with the SF_Cm(III)/Eu(III)_ ∼ 140 obtained in extraction experiments.^20^

#### Extraction Tests

**Figure 8:**
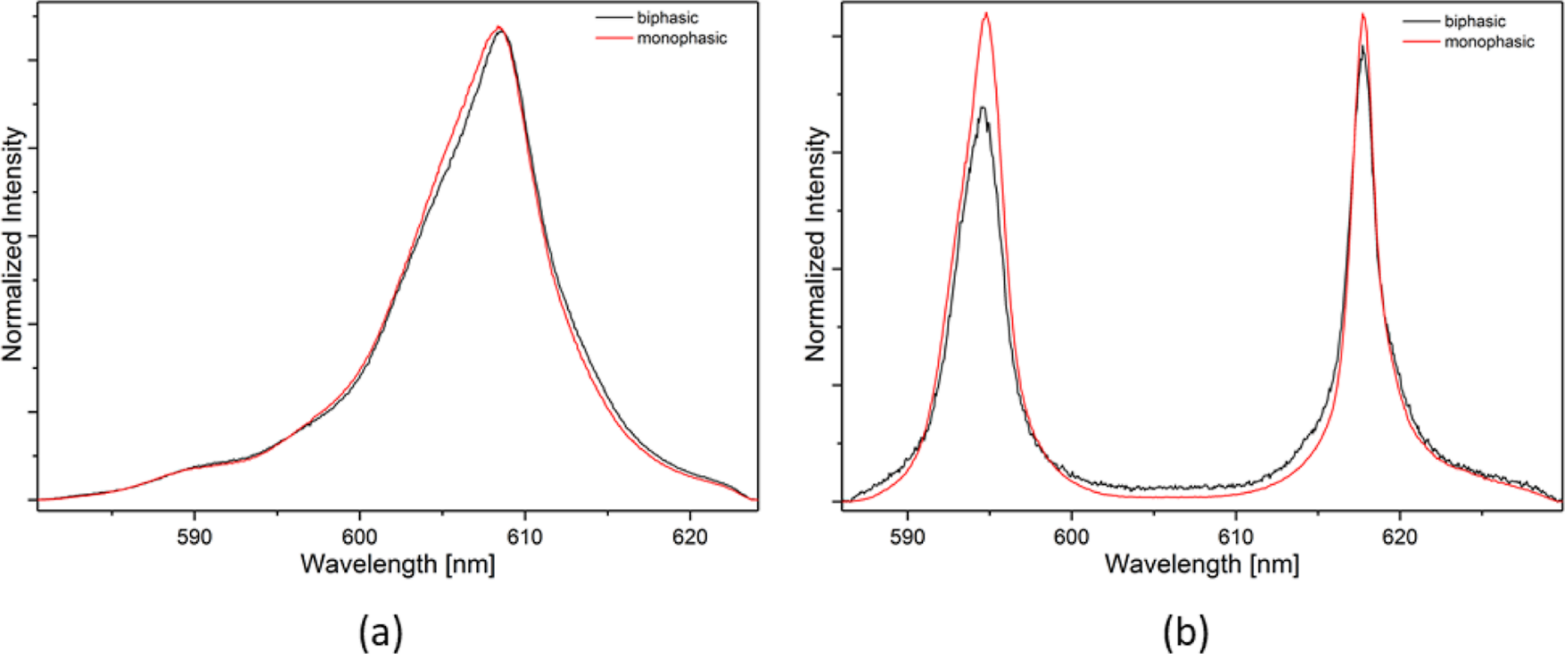
Comparison between the spectra of the organic phases from the extraction experiments and the monophasic solutions: [Cm(PTEH)_3_]^3+^ (a) and [Eu(PTEH)_3_]^3+^ (b), *T* = 20 °C.

To determine the stoichiometry of the Cm(III) and Eu(III) complexes involved in the extraction process, TRLFS analysis was performed on organic phases from extraction experiments. In Figure 8a, the fluorescence spectra of the [Cm(PTEH)_3_]^3+^ and in Figure 8b, the fluorescence spectra of the [Eu(PTEH)_3_]^3+^ are depicted together with the spectra from the respective organic solutions from the extraction experiments. Both for Cm(III) and Eu(III), spectra from monophasic and biphasic systems are in excellent agreement, proving both metals to be extracted as 1:3 complexes. Indeed, this study sheds light on the key role of the higher stoichiometry complexes also involved in the extraction process, in addition to what was demonstrated in previous studies by preliminary slope analysis.^18^

#### Fluorescence Lifetime

Fluorescence lifetime measurement is an additional way to follow the evolution of the Cm(III) and Eu(III) complexation with PTEH (Supporting Information). Each species can be identified by a typical lifetime related to the decay of the emission intensity. Figure S19 reports the decay of the emission intensity for the Cm(III) and Eu(III) solvent species. The observed lifetimes are τ = 94 ± 8 μs for Cm(III) and τ = 150 ± 12 μs for Eu(III), in agreement with the literature data.^28^

Moreover, the fluorescence lifetime was measured at the highest ligand concentration of the monophasic experiments as well as the organic phases of the extraction experiments. They are given in Table 4. Fluorescence lifetimes of both Cm(III) and Eu(III) increase drastically due to the replacement of the quenching solvent molecules (water and alcohol) further supporting the conclusion that [Cm(PTEH)_3_]^3+^ and [Eu(PTEH)_3_]^3+^ are the dominant species under extraction (biphasic) conditions.

**Table 4:** Fluorescence Lifetime τ of [M_Solv._]^3+^ and [M(PTEH)_3_]^3+^, with M = Cm and Eu in Monophasic and Biphasic Systems

	τ_Solvated_ [μs]	τ_Monophasic_ [μs]	τ_Biphasic_ [μs]
Cm(III)	94 ± 8	585 ± 12	405 ± 9
Eu(III)	150 ± 12	3643 ± 33	2557 ± 72

#### NMR: bonding of PTEH in Lu(III) and Am(III)-PTEH complexes

##### Lu-PTEH Complex Solution

In this study, the 1:3 Lu(III) complex was used as a diamagnetic reference. First characterization was performed by ^1^H NMR experiments (Figure S22). Peak assignment of the aromatic protons was accomplished: both the protons H_11_ (δ = 8*.*9 ppm) of the triazole moiety and H_5_ (δ = 8 ppm) of the pyridine ring are weakly shifted downfield with respect to the free ligand, whereas a more pronounced shift in the same direction is observed for the proton H_4_ (δ = 8*.*3 ppm) of the central pyridine. Peak assignment was confirmed by 2D heteronuclear correlation spectroscopy; a ^1^H,^15^N-HMQC spectrum of the [Lu(PTEH)_3_](OTf)_3_ complex was obtained and reported (Figure S23). The 2D plot shows correlations of the protons in the ethylhexyl side-chain H_12_ to the non-coordinating nitrogen atom N_9_ (δ = 365 ppm) of the triazole moiety; the signals H_4_ and H_3/5_ correlate to the coordinating nitrogen atom N_1_ (δ = 268 ppm) of the pyridine moiety, whereas the proton H_11_ reveals correlations to the coordinating nitrogen atom N_8_ (δ = 317 ppm) of the triazole moiety and to the non-coordinating nitrogen atoms N_10_ (δ = 254 ppm) and N_9_. Figure S24 shows a direct overlay of the ^1^H,^15^N-HMQC spectra of the free ligand and Lu(III) complex.

##### Am-PTEH Complex Solution

The 1:3 complex was characterized by 1D and 2D NMR. The corresponding spectra are reported in the Supporting Information. Only one set of new signals can be identified, indicating that only one complex species is present in the solution, which is supported by ^1^H diffusion ordered spectroscopy (see Figure S28) as well. Backed up by 2D heteronuclear correlations, all protons except for the overlapping signals in the side chains were assigned. The singlet H_11_ (δ = 8*.*9 ppm) appears to be shifted downfield compared to the corresponding free ligand signal (δ = 8*.*6 ppm), whereas the doublet H_3/5_ (*δ* = 7*.*8 ppm) and the triplet H_4_ (δ = 7*.*6 ppm) appear to be shifted upfield with respect to the equivalent free ligand peak (δ = 7*.*9 ppm).

**Figure 9:**
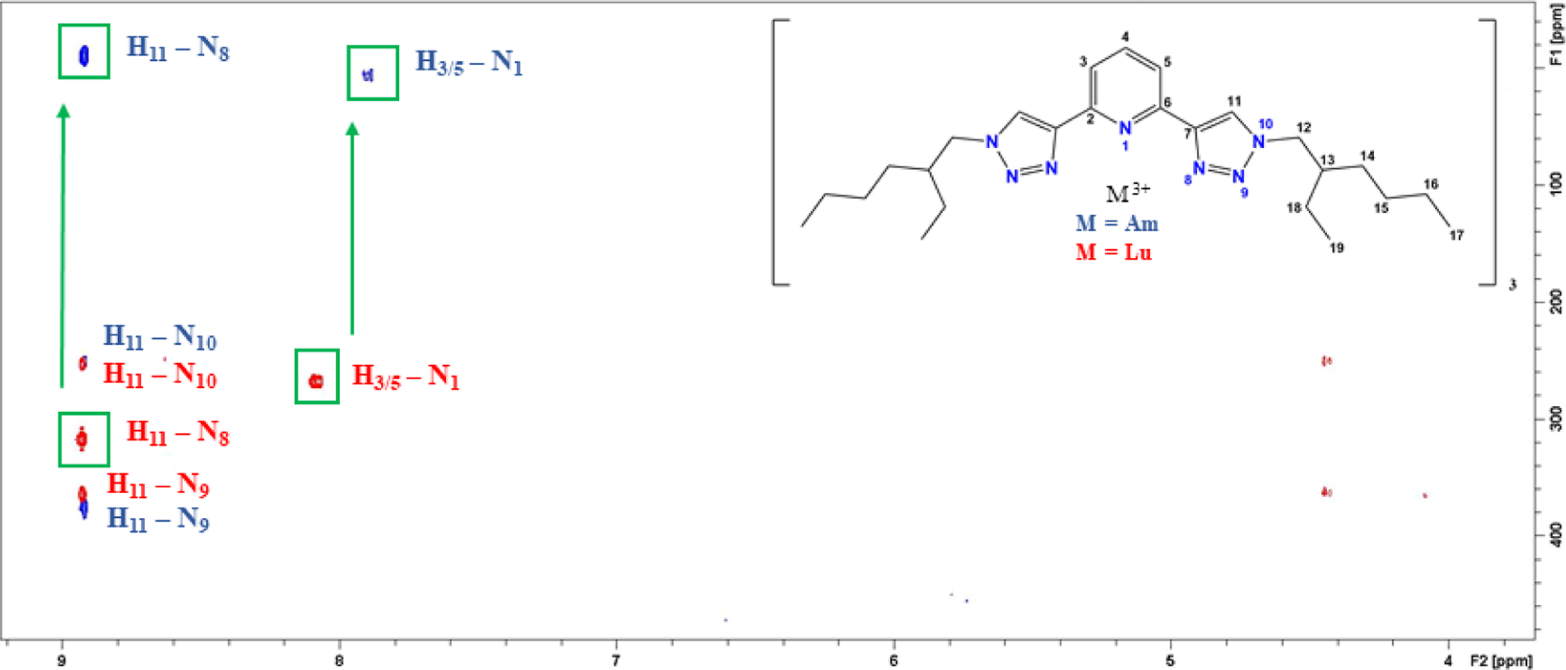
Overlay of two ^1^H,^15^N-HMQC spectra of [Am(PTEH)_3_](OTf)_3_ complex and [Lu(PTEH)_3_](OTf)_3_. Correlations of the Am(III) complex are in blue, whereas those of Lu(III) are shown in red.

^1^H,^15^N-HMQC experiments were performed for the 1:3 [Am(PTEH)_3_](OTf)_3_ complex in pure CD_3_OD and compared to the corresponding Lu(III) complex (Figure 9). A comparison to the free ligand is given in the Supporting Information. In comparison to the Lu(III) complex, strong upfield shifts (260 ppm for N_1_ and 330 ppm for N_8_) of the coordinating nitrogen atoms are observed for the Am(III) complex while the non-coordinating nitrogen atoms N_9_ and N_10_ are barely shifted.

The shifts of the coordinating nitrogen atoms can be caused by the paramagnetism of Am(III) or a different bonding between the ligand and Am(III), namely, a higher covalent bond character. In fact, similar strong shifts of coordinating nitrogen atoms have been found for Am(III) complexes with other *N*-donor ligands, in which a higher covalent bond character was responsible for the observed shift of the coordinating nitrogen atoms.^23,24^

To study the paramagnetic contribution on the strong shifts of the coordinating nitrogen atoms, temperature-dependent ^1^H,^15^N-HMQC spectra of [Am(PTEH)_3_](OTf)_3_ were recorded between 245 and 325 K (see Figures S33, S34, and S35). Shifts of up to 5 ppm for the coordinating nitrogen donor atoms were found in the studied temperature range, proving the weak paramagnetism of Am(III) complexes as stated in the literature.^29^ Therefore, the strong shift of the nitrogen donor atoms in the Am(III) complex does not result from the paramagnetism of Am(III) but is evidence for a higher covalent bond character in the Am(III)–N bond. This higher covalent bond character which only exists in An(III) but not in Ln(III) complexes is the reason for the ligand’s selectivity for An(III).

## Conclusions

The present work aimed to deepen the understanding of the complexation behavior of 2,6-bis(2-ethylhexyl-1H-1,2,3-triazol-4-yl)pyridine (PTEH), whose promising extracting performances have made it a potential candidate for a regular-SANEX process to extract An(III) downstream of the DIAMEX process, or for an advanced 1c-SANEX process to separate them directly from the PUREX raffinate. The first insight into the metal/ligand stoichiometry of the species formed upon complexation with PTEH was obtained by ESI mass spectrometry. Speciation spectra of La(III) and Eu(III) nitrates with PTEH in monophasic solutions containing HNO_3_ showed the formation of the positive mono charged 1:2 [ML_2_(NO_3_)_2_]^+^ complex as the dominant species, along with a small contribution of the 1:3 [ML_3_(NO_3_)]^2+^ complex, even though the 1: 3 contribution appears more pronounced for Eu(III). Moreover, a series of experiments without HNO_3_ demonstrated the negligible influence of acidity on the complexation mechanism involved. Complexation of La(III) and Eu(III) nitrates with PTEH was investigated in more real process-like conditions by biphasic solutions ([*L*]/[*M*] = 1). ESI–MS analyses on the organic phase upon extraction tests showed a good agreement with the results coming from the monophasic solutions at the same *M*/*L* ratio. Indeed, the 1:2 [LaL_2_(NO_3_)_2_]^+^ complex appeared as the most intense species involved in the extraction process along with the less intense 1:3 [LaL_3_(NO_3_)]^2+^ complex.

Moreover, the complexation of Cm(III) and Eu(III) with PTEH was investigated by TRLFS. Fluorescence analyses of monophasic solutions revealed three complex species [M(PTEH)_*n*_]^3+^ (*n* = 1–3) for both Cm(III) and Eu(III). A difference of 2 orders of magnitude between the stability constants of the 1:3 [Cm(PTEH)_3_]^3+^ and [Eu(PTEH)_3_]^3+^ complex species was observed. These results led to a separation factor SF_Cm(III)/Eu(III)_ = 126 ± 4, thereby confirming the actinide over lanthanide selectivity of this *N*-donor ligand derived from the extraction data. TRLFS was also applied to identify the major species formed in the organic phase upon solvent extraction experiments. Comparison of the fluorescence spectra from the organic phase samples with those of the titration experiments appeared in good agreement for both Cm(III) and Eu(III), thereby confirming the formation of the [Cm(PTEH)_3_]^3+^ and [Eu(PTEH)_3_]^3+^ complexes during extraction.

Finally, insights into the bonding of Ln(III) and An(III) with PTEH were gained by NMR spectroscopy to better understand the ligand’s selectivity toward actinides. The 1:3 [Lu(PTEH)_3_](OTf)_3_ and [Am(PTEH)_3_](OTf)_3_ complexes were characterized, identifying all potential chemical shifts upon complexation compared to free ligand spectra by 2D heteronuclear correlation experiments. The most important findings were produced by the overlay of the ^1^H,^15^N-HMQC spectra for Lu(III) and Am(III) complexes. Tremendous chemical shift differences of the coordinating nitrogen atoms exist between the Lu(III) complex and the Am(III) complex that cannot be explained by a pure contribution of paramagnetism but are evidence for a higher covalent bond character in the Am(III) complex being the driving force of the ligand’s selectivity.

## Supporting Information Available

ESI–MS experimental details [ESI–MS spectra of the components; La(III) speciation with PTEH; kinetic stability of La(III)-PTEH species; and Eu(III) speciation with PTEH]; TRLFS experimental details [complexation of Cm(III) with PTEH; complexation of Eu(III) with PTEH; and fluorescence lifetime measurements] and NMR experimental details [bonding of PTEH with Lu(III) and Am(III) and temperature-dependent experiments] (PDF)
