# Supplementary material for: Insights into the Complexation Mechanism of a Promising Lipophilic PyTri Ligand for Actinide Partitioning from Spent Nuclear Fuel
Source: Inorg Chem. 2022 Nov 4;61(46):18400–11. doi: 10.1021/acs.inorgchem.2c02332 (PMC9682475; doi:10.1021/acs.inorgchem.2c02332)
Supplement: Supplementary file 1 — ic2c02332_si_001.pdf [file ic2c02332_si_001.pdf]

SUPPORTING INFORMATION

Insights into the complexation mechanism of a promising lipophilic PyTri ligand for actinide partitioning from spent nuclear fuel

Francesco Galluccio,[a] Elena Macerata,[*,a] Patrik Weßling,[*,b,c] Christian Adam,[b] Eros Mossini,[a] Walter Panzeri,[d] Mario Mariani,[a] Andrea Mele,[d,e] Andreas Geist,[b] and Petra J. Panak,[b,c]

[a]Politecnico di Milano, Department of Energy, Piazza Leonardo da Vinci 32, 20133 Milano, Italy

[b]Karlsruhe Institute of Technology (KIT), Institute for Nuclear Waste Disposal (INE), P.O. Box 3640, 76021 Karlsruhe, Germany

[c]Heidelberg University, Institute for Physical Chemistry, Im Neuenheimer Feld 253, 69120 Heidelberg, Germany

[d]C.N.R. – Consiglio Nazionale delle Ricerche, Istituto di Scienze e Tecnologie Chimiche "G. Natta" (SCITEC), Sezione "U.O.S. Milano Politecnico", Milan, Italy

[e]Politecnico di Milano, Department of Chemistry, Materials and Chemical Engineering "G. Natta", Piazza Leonardo da Vinci 32, 20133, Milano, Italy

Corresponding authors: elena.macerata@polimi.it, patrik.wessling@partner.kit.edu

Summary

A. ESI-MS study 2

ESI-MS spectra of the components 2

La(III) speciation with PTEH 3

Eu(III) speciation with PTEH .... **Errore. Il segnalibro non è definito.**

Kinetic stability of La(III)- PTEH species 6

Eu(III) speciation with PTEH 8

B. TRLFS study 10

Complexation of Cm(III) with PTEH 10

Complexation of Eu(III) with PTEH 11

Fluorescence lifetime measurements 12

C. NMR study 13

Bonding of PTEH with Lu(III) and Am(III) 13

Temperature-dependent experiments 19

A. ESI-MS study

ESI-MS spectra of the components

The pure PTEH spectrum was recorded in the positive ion mode (+MS), with a mass range from m/z 100 to 1200 and with a target mass at m/z 500. A cone voltage of 40 V was used according to the experimental standard conditions.

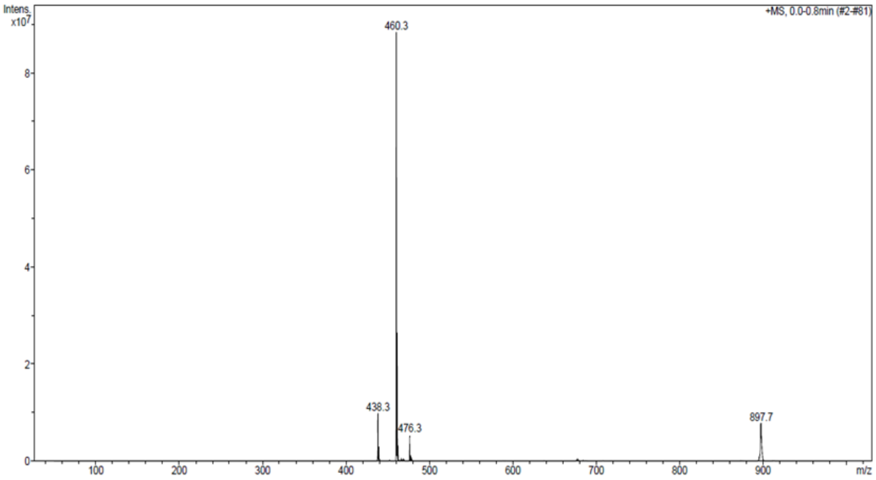

Figure S1: Positive ESI-MS spectrum of the pure ligand PTEH dissolved in kerosene-1-octanol 10%, diluted in acetonitrile.

Peak identification was first performed by collision-induced dissociation by isolating the precursor ion and varying the fragmentation voltage, secondly a comparison with the calculated isotopic pattern was made for the most prominent peaks.

Table S1: Major PTEH adducts in the pure ligand spectrum.

m/z	Species
**438.3**	$[H(PTEH)]^+$
**460.3**	$[Na(PTEH)]^+$
**476.3**	$[K(PTEH)]^+$
**897.7**	$[Na(PTEH)_2]^+$

In order to observe the La(III) nitrate species, the spectrum was recorded in the negative ion mode (-MS) in the 100 to 1200 mass range and with a target mass of 500 m/z.

As shown in Figure S2, the most intense signal belongs to the species $[La(NO_3)_4]^-$, which has to be taken into account in view of the further speciation studies upon extraction tests. At increasing mass over charge ratio, more and more nitrate clusters coordinating La^{3+} and Na^+ cations are clearly visible in the spectrum. The Collision Induced Dissociation (CID) of the selected peak at m/z 711.6 leads to one more detectable signal at lower m/z by losing a La(III) and three nitrate ions, thereby giving the species $[La(NO_3)_4]^-$.

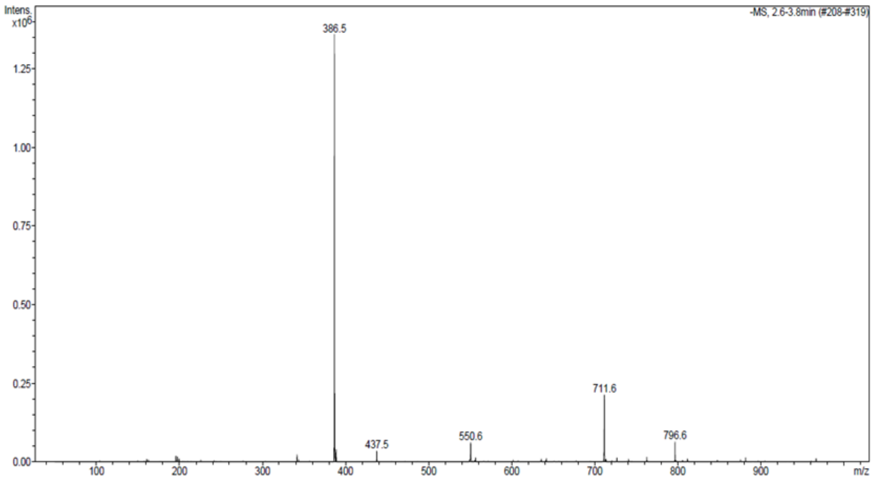

Figure S2: Negative ESI-MS spectrum of the pure La(III) nitrate in acetonitrile.

Table S2: Peak assignment of the major La(III) nitrate species.

m/z	Species
**386.5**	$[La(NO_3)_4]^-$
**711.6**	$[La_2(NO_3)_7]^-$
**796.6**	$[La_2Na(NO_3)_8]^-$

La(III) speciation with PTEH

The different monophasic solutions were prepared by mixing suitable volumes of 10^{-3} M ligand stock solution and 10^{-3} M La(III) nitrate stock solution containing 3 M HNO_3 and then diluting the mixture up to suitable values for infusion into the mass spectrometer source. In turn, the PTEH and the La(III) nitrate stock solutions were obtained by diluting the corresponding mother solutions prepared according to the extraction conditions.

An equimolar solution was prepared by adding 10 μL of 0.2 M PTEH mother solution and 10 μL of 0.2 M La(III) nitrate solution, subsequently diluted in acetonitrile to 10^{-4} M. Spectra were recorded in positive ion mode at ratio [L]/[M] equal to 1.

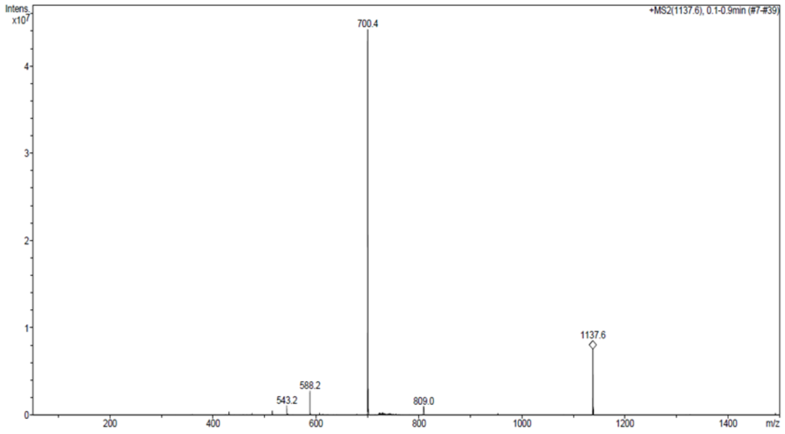

Figure S3: MS^2 spectrum of the complex species at m/z 1137.5; the major fragment at m/z 700.4 represents the $[La(NO_3)_2L]^+$ species, revealing the loss of one ligand molecule upon fragmentation.

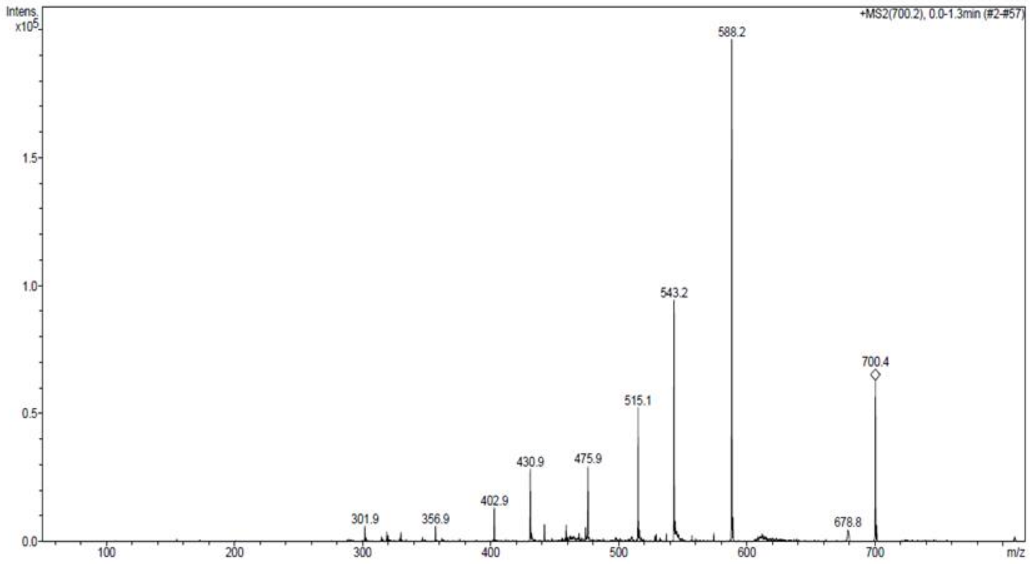

Figure S4: MS^2 spectrum of the 1:1 $[La(NO_3)_2L]^+$ complex species at m/z 700.4; most of fragments are in common with the previous MS^2 spectrum.

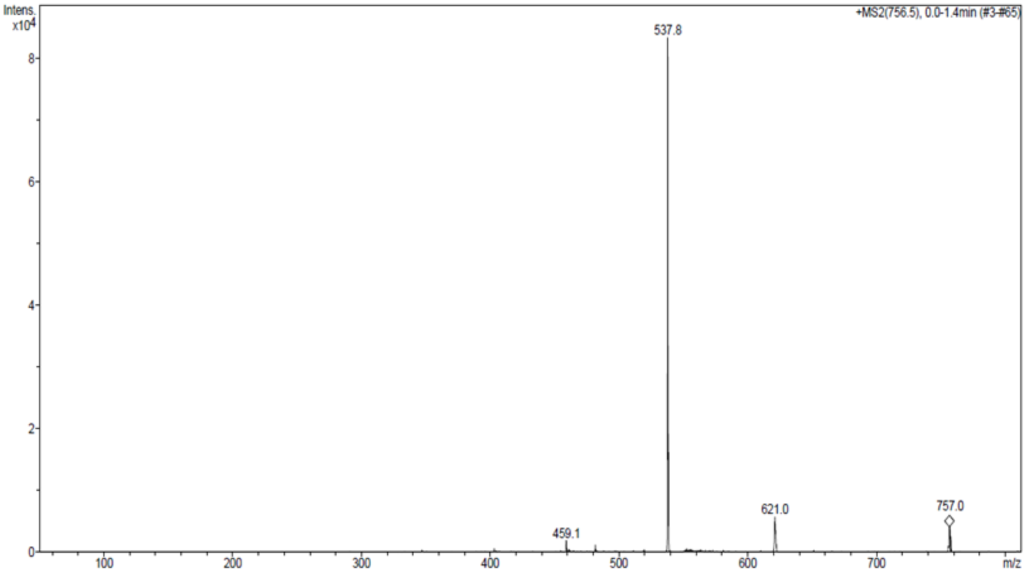

Figure S5: Fragmentation spectrum of the isolated peak at m/z 757.

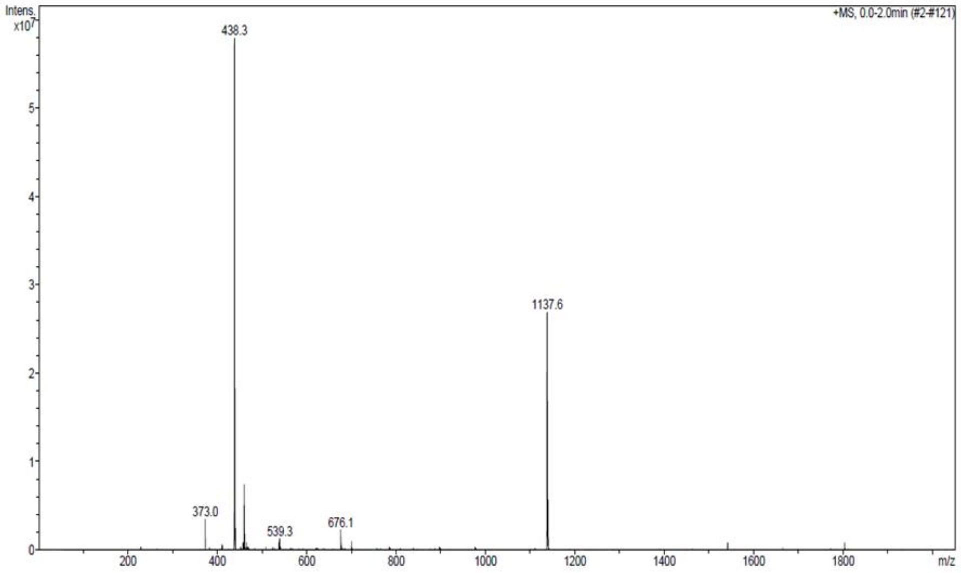

Figure S6: Positive ESI-MS spectrum of the equimolar solution containing La(III) nitrate and PTEH in acetonitrile without nitric acid.

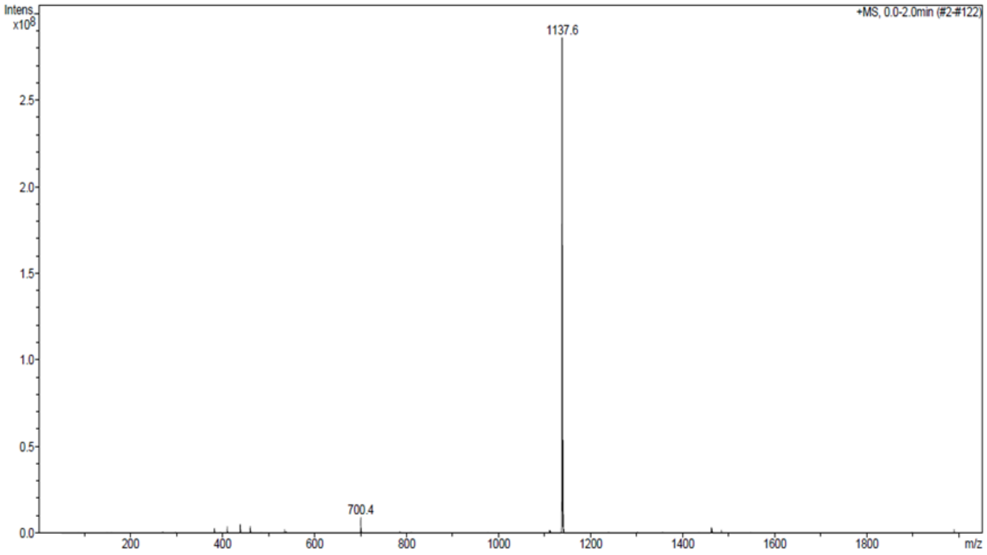

Figure S7: High mass positive ESI-MS spectrum of solutions containing La(III) nitrate and PTEH at [L]/[M] ratio equal to 5 in acetonitrile without nitric acid.

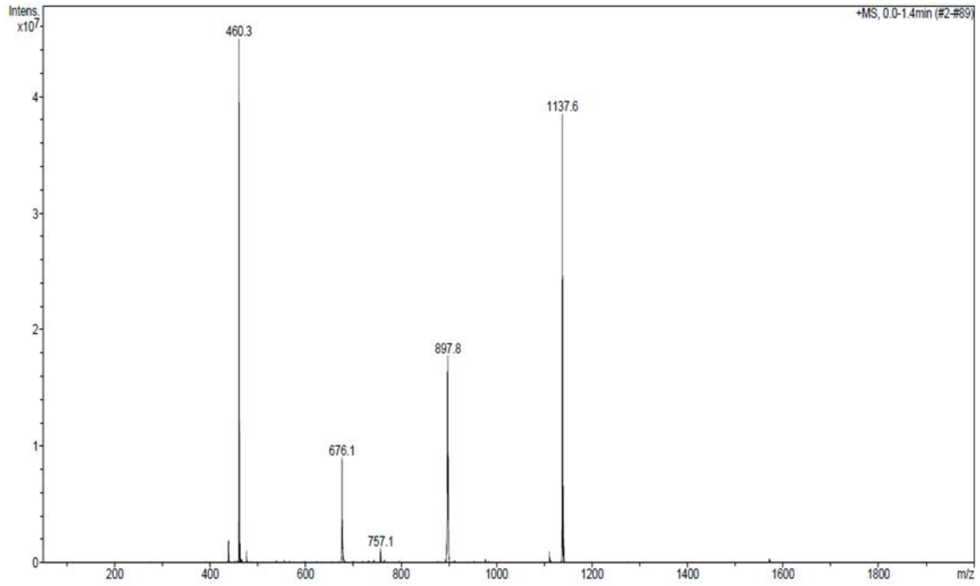

Figure S8: Positive ESI-MS spectra of solutions containing La(III) nitrate and PTEH at [L]/[M] ratio equal to 10 in acetonitrile without nitric acid.

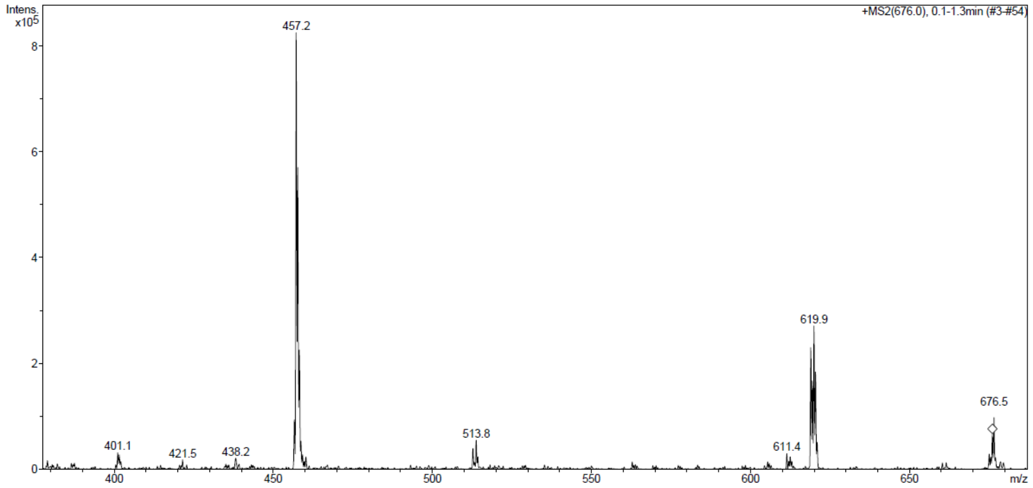

Figure S9: Fragmentation spectrum of the isolated peak at m/z 676.5.

Kinetic stability of La(III)- PTEH species

Kinetic stability of the gas phase La(III) nitrate complexes with PTEH was explored by means of CID. First, collisions were carried out in the electrospray ionization source (in-source CID) by varying the cone voltage, then the standard CID in the ion trap was performed thus obtaining the fragmentation spectra of the 1:2 and 1:3 major complexes. Collision induced dissociation can be carried out in the interface region between the ionization source at atmospheric pressure and the single quadrupole mass analyser or ion trap. By varying the cone voltage, energy-resolved mass spectra can be obtained leading to breakdown graphs which represent the energy dependence of the ion abundance. In-source CID is a very simple way to get qualitative information about the energy involved in the fragmentation pathways. Before performing any speciation study by ESI-MS, it is crucial to optimize the cone voltage as a function of m/z to detect all components in a mixture. [1] The breakdown curves represent the ion abundance of the major complexes of the parent spectrum as a function of the cone voltage. [2] The ion abundance of some complexes slightly goes down at higher cone voltage thereby

showing the ion decomposition due to an increased internal energy. Using 40 V as standard cone voltage could be considered adequate to perform ESI-MS speciation analysis and obtain a good ion abundance.
Instead of multiple collisions like the in-source CID process due to the high curtain gas pressure, the CID is performed by single collisions in the spectrometer ion trap where the gas pressure is lower. The CID was also exploited several times in these experimental activities to confirm the peak assignments of each parent spectrum. First, the interested peak of the spectrum is isolated, then the peak dissociation is performed thereby obtaining a fragmentation pattern. Figure S10 shows an example of the preferential fragmentation pathway consisting of a detachment of a PTEH molecule rather than a nitrate counterion. The fragmentation pathway observed for the major complex $[La(NO_3)_2L_2]^+$ is described by Equation (1).

$$[La(NO_3)_2PTEH_2]^+ \leftrightharpoons [La(NO_3)_2PTEH]^+ + PTEH \qquad (1)$$

Moreover, the fragmentation pathway of the 1:3 complex $[La(NO_3)L_3]^{2+}$ confirms the loss of a PTEH molecule following the CID process as shown by Equation (2).

$$[La(NO_3)PTEH_3]^{2+} \leftrightharpoons [La(NO_3)PTEH_2]^{2+} + PTEH \qquad (2)$$

Following the previous experimental activities, some relevant remarks could be pointed out about a weak PTEH binding affinity to La(III) in the presence of nitrate ions in the gas-phase complexes. Good evidence of this could be found in the CID of some complexes free of nitrate ions. The preferential loss of one PTEH molecule is replaced with just a partial fragmentation thereby confirming the influence of nitrates on ligand affinity towards La(III) and on complex stability as well, as observed in literature. [3]

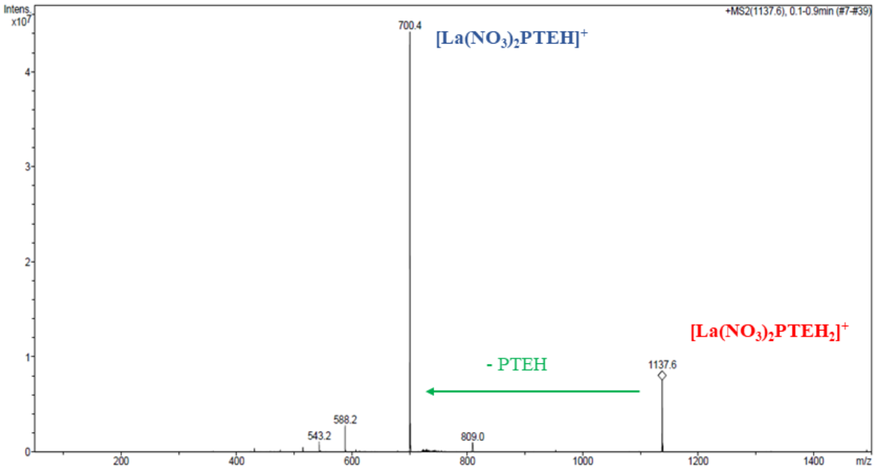

Figure S10: Example of the recurrent fragmentation pathway of the major PTEH complexes.

Eu(III) speciation with PTEH

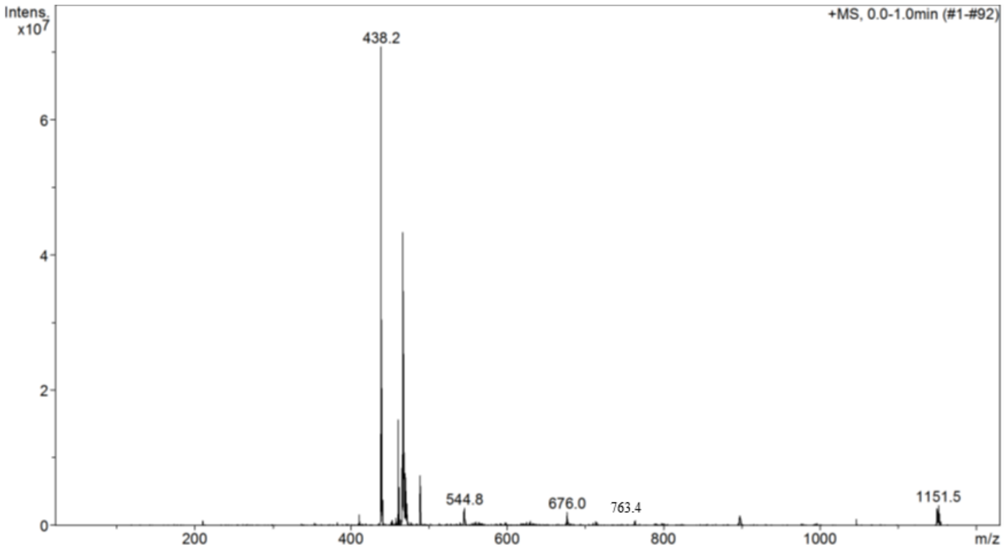

Figure S11: Positive ESI-MS spectrum of the equimolar solution containing Eu(III) nitrate and PTEH in acetonitrile with 3M HNO_3.

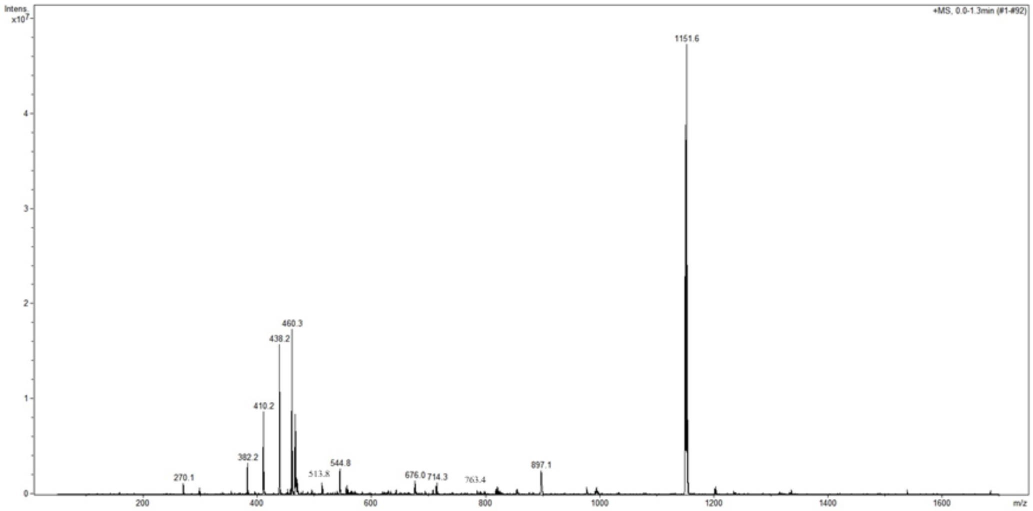

Figure S12: High mass positive ESI-MS spectrum of the equimolar solution containing Eu(III) nitrate and PTEH in acetonitrile with 3M HNO_3.

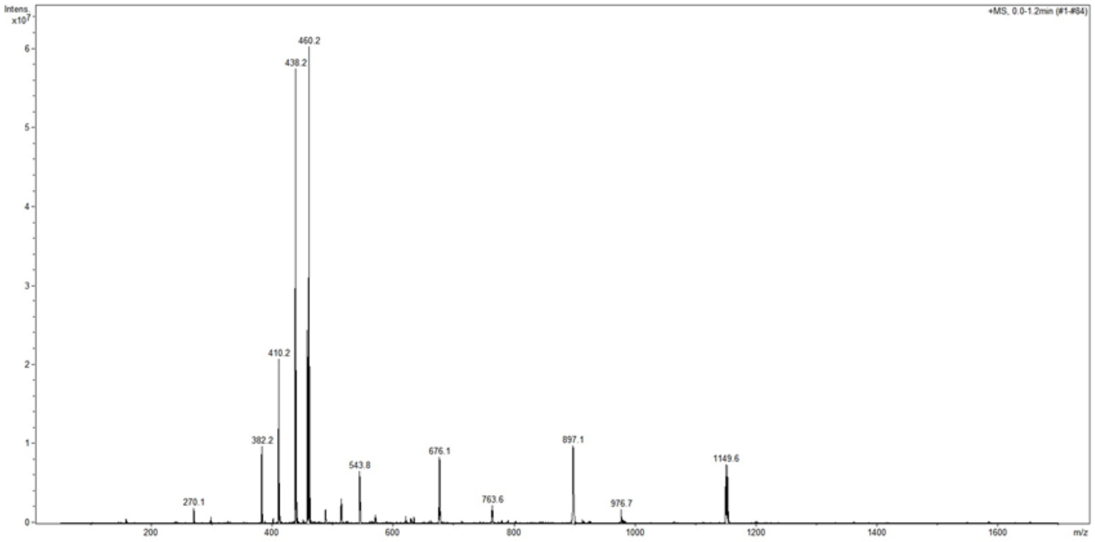

Figure S13: High mass positive ESI-MS spectrum of the solutions containing Eu(III) nitrate and PTEH at [L]/[M] ratio equal to 10 with 3 M HNO_3.

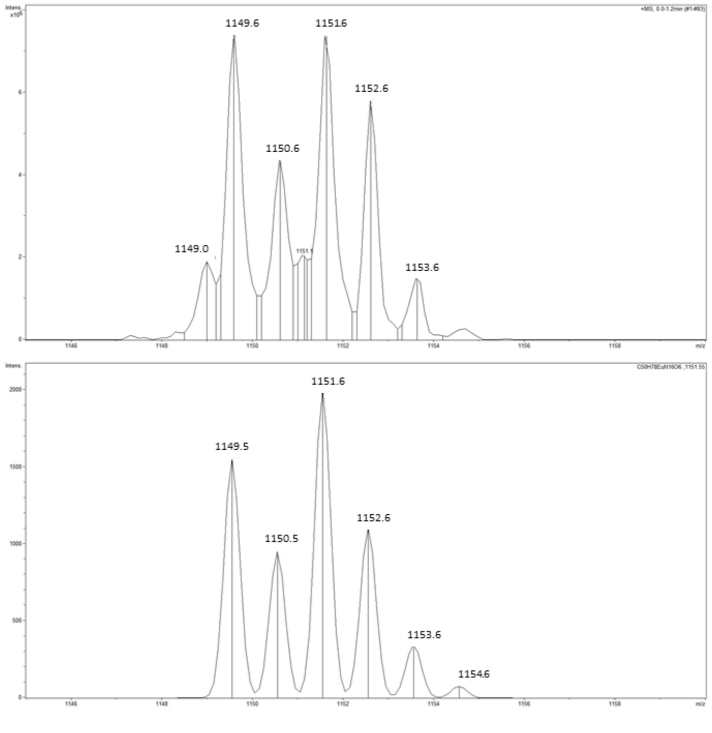

Figure S14: Comparison between the experimental spectrum (*top*) and the calculated isotopic pattern (*bottom*) of the 1:2 $[Eu(NO_3)_2L_2]^+$ complex species at m/z 1151.6.

B. TRLFS study

Complexation of Cm(III) with PTEH

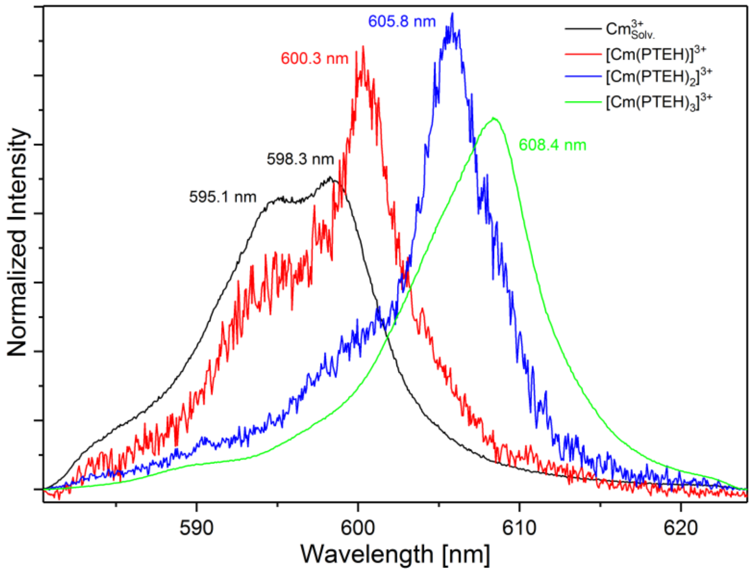

Figure S15: Normalized single component spectra of the Cm(III) solvent species and the $[Cm(PTEH)_n]^{3+}$ (n = 1 − 3) complexes in methanol with 5 vol% H_2O.

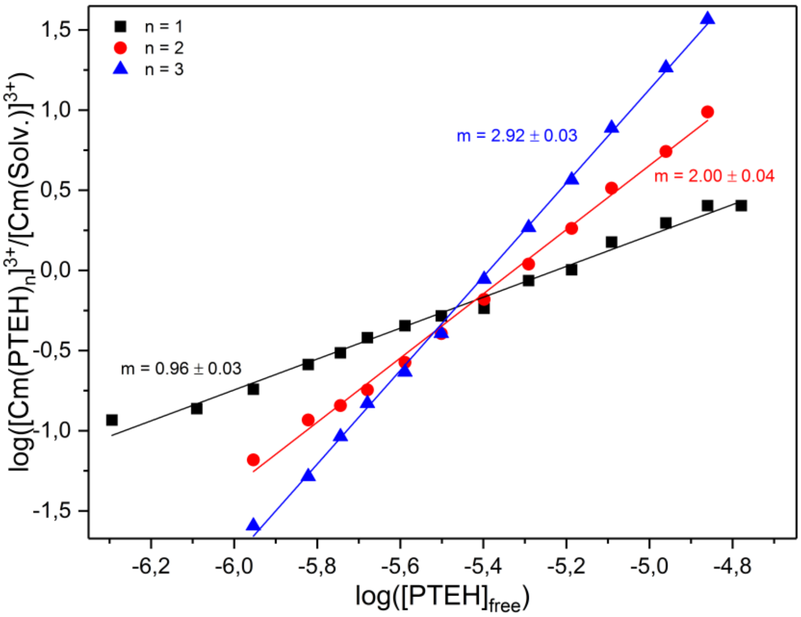

Figure S16: Double logarithmic plot of the concentration ratio $[Cm(PTEH)_n]^{3+}/[Cm_{Solv.}]^{3+}$ and the free ligand concentration.

Complexation of Eu(III) with PTEH

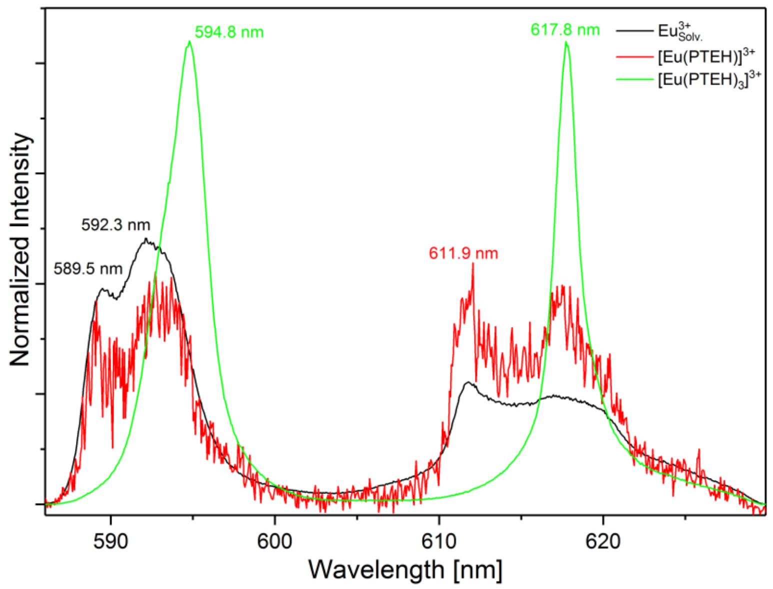

Figure S17: Normalized single component spectra of the $[Eu_{Solv.}]^{3+}$ species and the $[Eu(PTEH)_n]^{3+}$ (n = 1 − 3) complexes in methanol with 5 vol% H_2O.

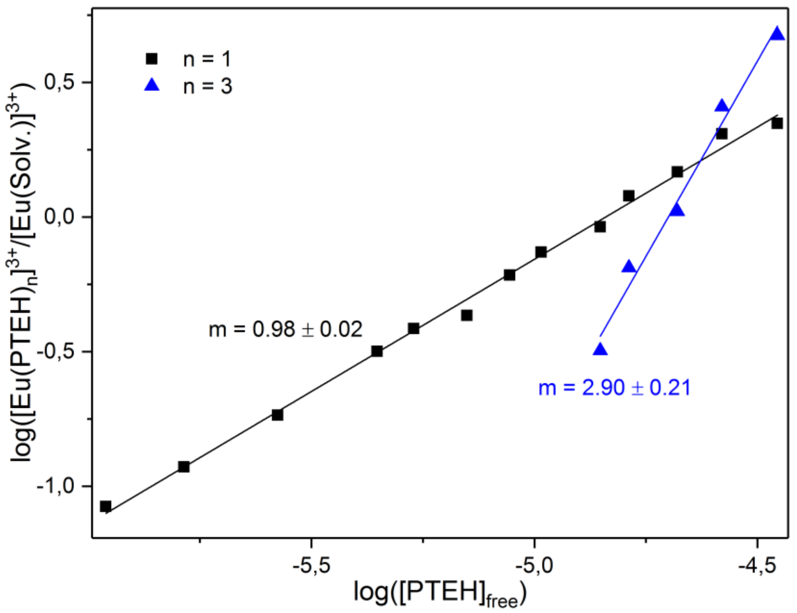

Figure S18: Double logarithmic plot of the concentration ratio $[Eu(PTEH)_n]^{3+}/[Eu_{Solv.}]^{3+}$ and the free ligand concentration.

Fluorescence lifetime measurements

The emission intensity decay was scanned at increasing delay time by 20 µs for 50 steps. The lifetime τ was calculated by fitting the fluorescence intensity as a function of the delay time after the laser pulse.

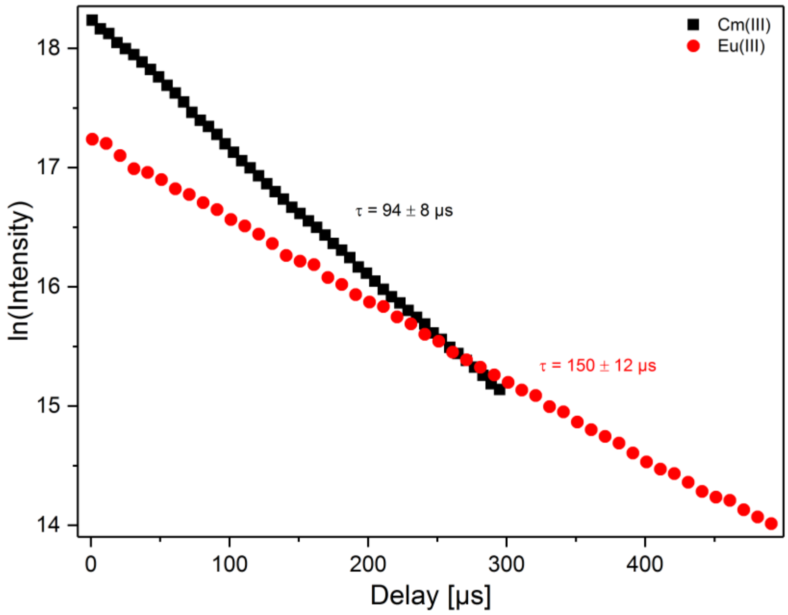

Figure S19: Logarithmic fluorescence intensity decay of the solvated Cm(III) and Eu(III) ions in methanol with 5 vol% H_2O.

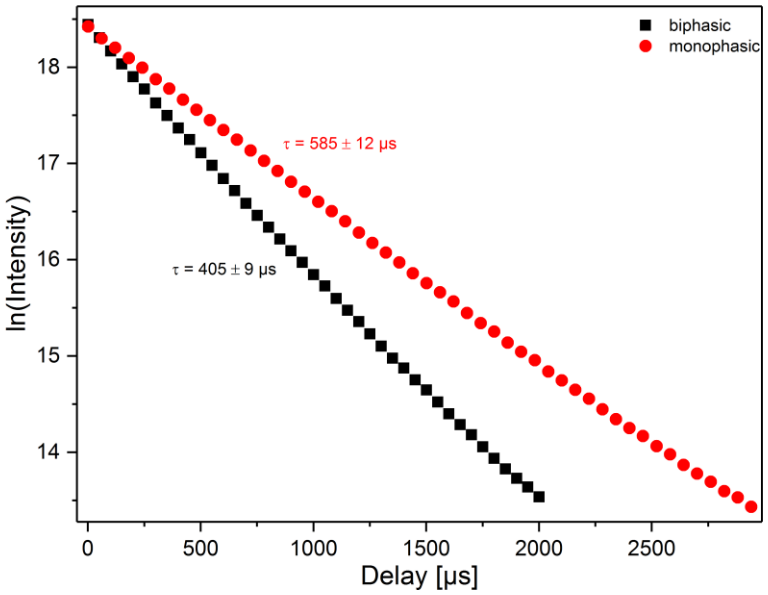

Figure S20: Logarithmic fluorescence intensity decay of the $[Cm(PTEH)_3]^{3+}$ complex in biphasic and monophasic solutions (highest PTEH concentration of the titration experiment).

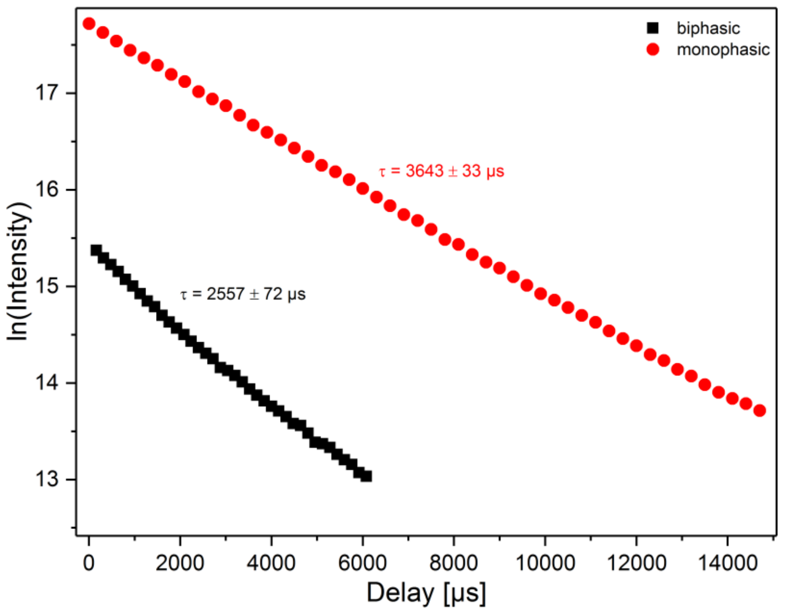

Figure S21: Logarithmic fluorescence intensity decay of the $[Eu(PTEH)_3]^{3+}$ complex in biphasic and monophasic solutions (highest PTEH concentration of the titration experiment).

C. NMR study

Bonding of PTEH with Lu(III) and Am(III)

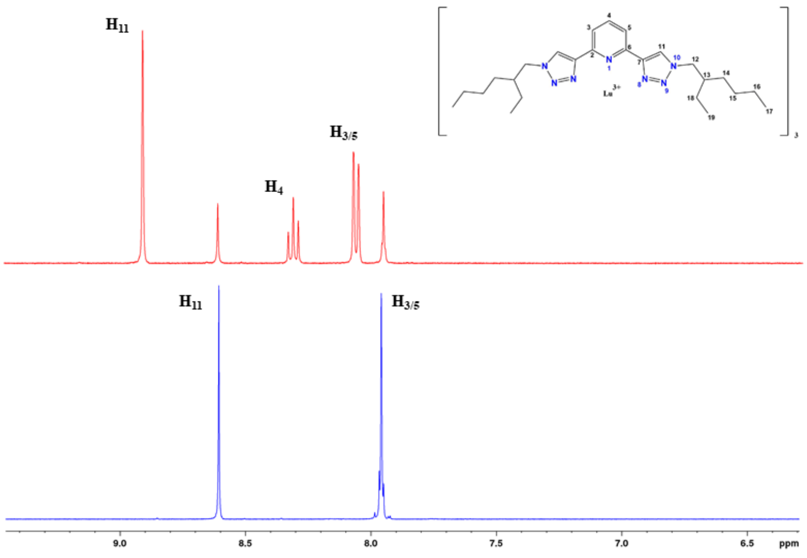

Figure S22: Stacked plot of the aromatic region for the stoichiometric reaction of PTEH with $Lu(OTf)_3$ in CD_3OD. 1D ^1H NMR spectrum of the 1:3 $[Lu(PTEH)_3](OTf)_3$ complex (*top*). Free ligand spectrum of 0.03 M PTEH solution (*bottom*).

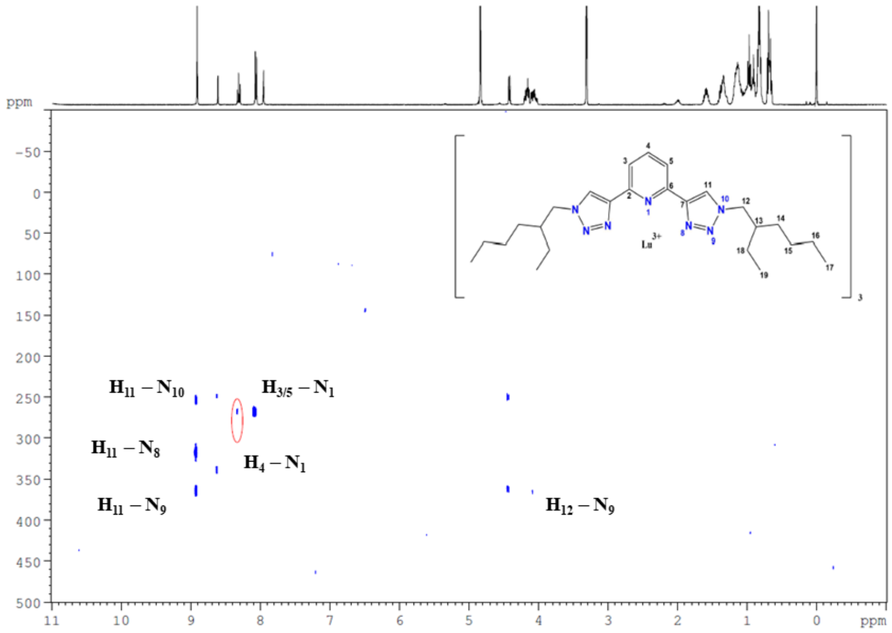

Figure S23: $^{1}H,^{15}N$ HMQC spectrum of the $[Lu(PTEH)_3](OTf)_3$ complex in CD_3OD.

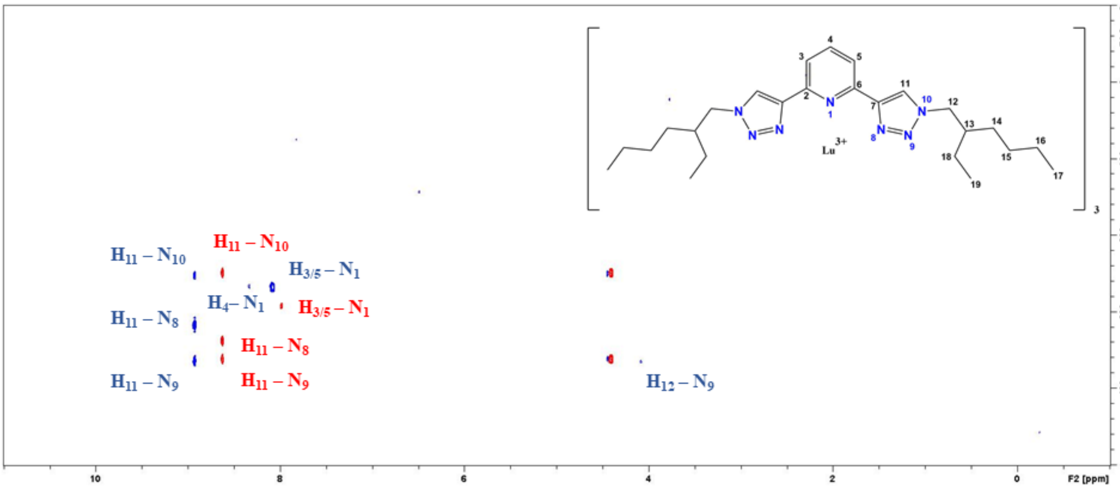

Figure S24: Overlay of two ^{1}H, ^{15}N HMQC spectra of $[Lu(PTEH)_3](OTf)_3$ complex and free ligand. Correlations of the Lu(III) complex are in blue, whereas those of the free ligand arein red.

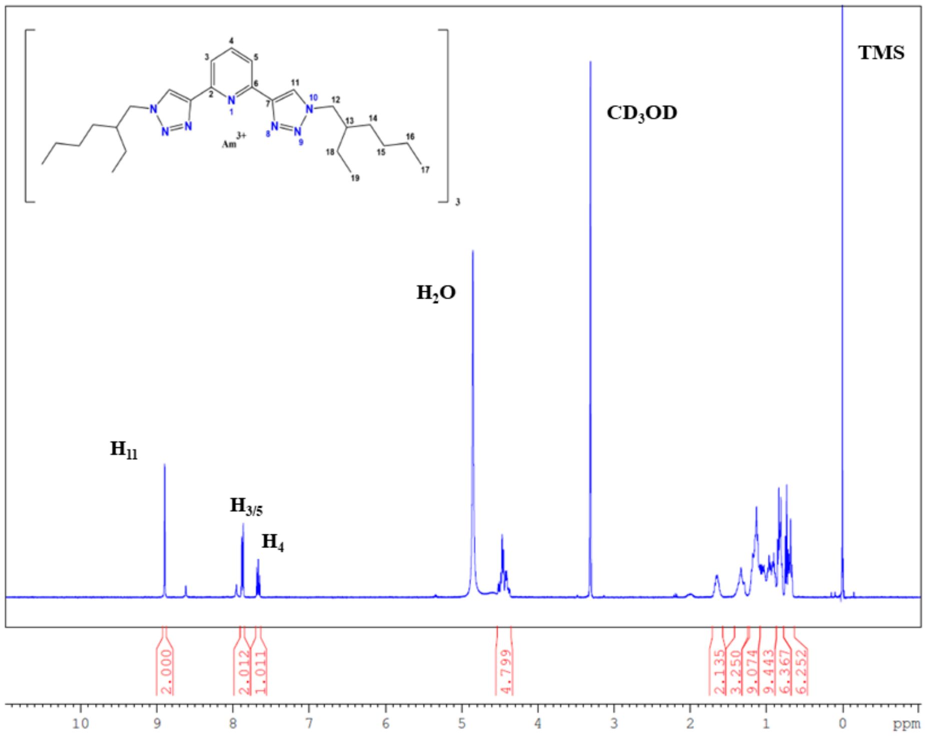

Figure S25: 1D ^{1}H NMR spectrum of the $[Am(PTEH)_3](OTf)_3$ complex in CD_3OD.

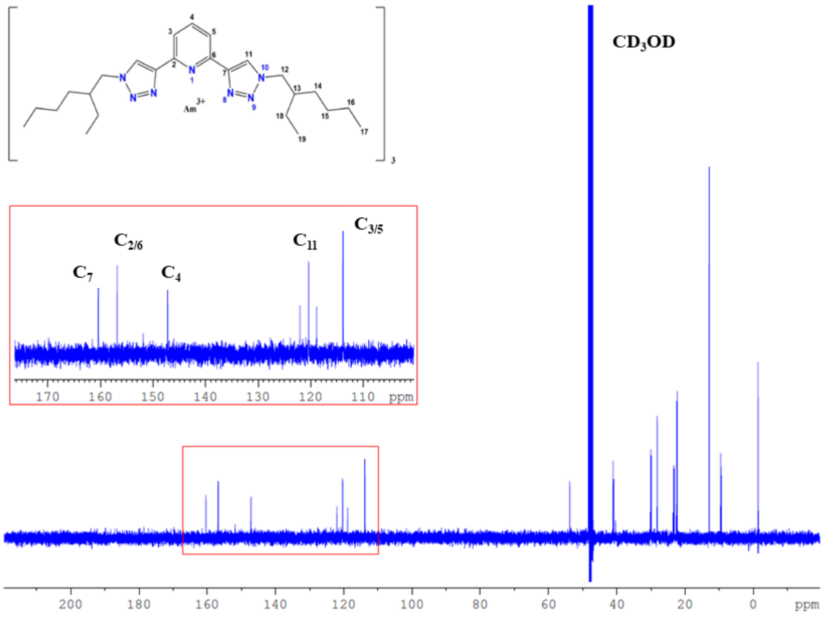

Figure S26: 1D ^{13}C NMR spectrum of the $[Am(PTEH)_3](OTf)_3$ complex in CD_3OD. An expansion of the aromatic region of the spectrum is outlined in red.

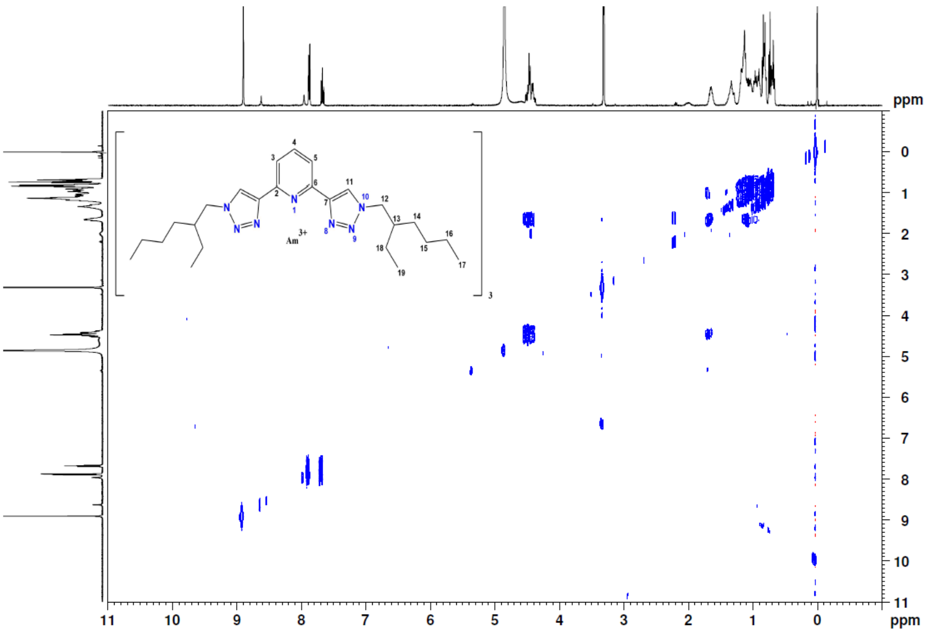

Figure S27: ^{1}H COSY spectrum of the $[Am(PTEH)_3](OTf)_3$ complex in CD_3OD. Both the horizontal and vertical axis show the ^{1}H NMR chemical shifts.

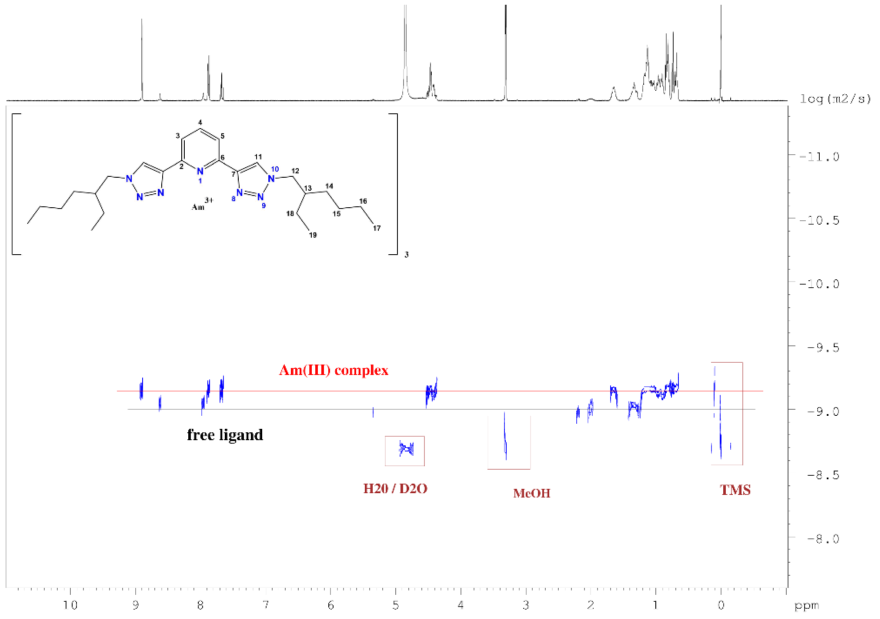

Figure S28: ^{1}H-DOSY spectrum of the 1:3 Am(III) complex in CD_3OD. ^{1}H chemical shifts are reported on the horizontal axis, whereas the logarithmic diffusion coefficients are reported on the vertical axis. The aligned signals representing each single complex are marked in blue.

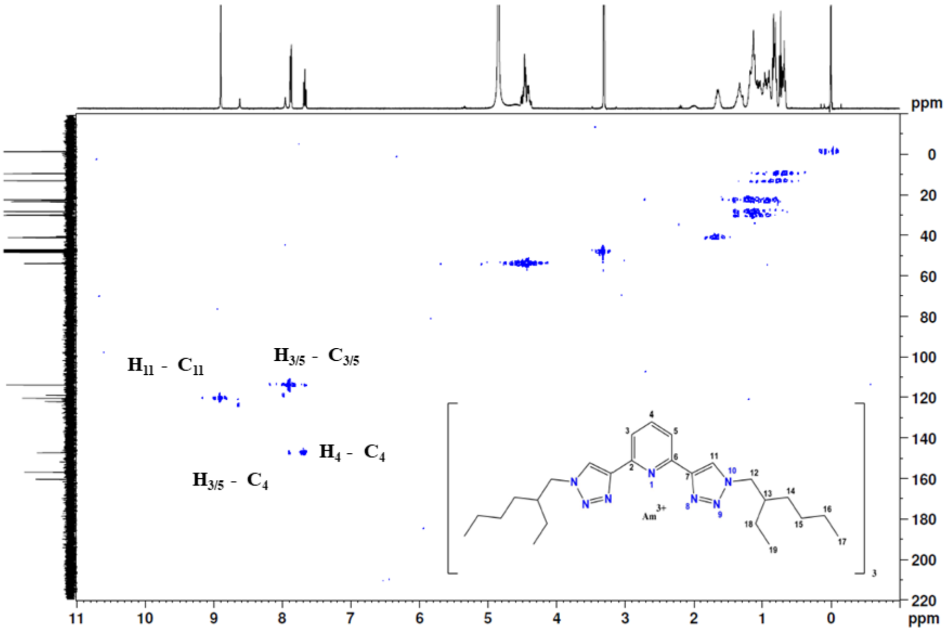

Figure S29: $^{1}H,^{13}C$ HSQC spectrum of the $[Am(PTEH)_3](OTf)_3$ complex in CD_3OD.

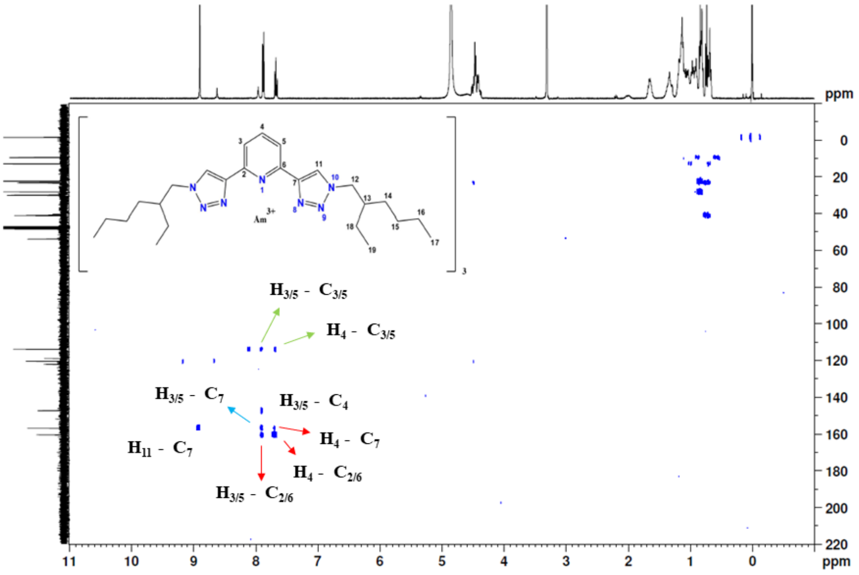

Figure S30: $^{1}H,^{13}C$ HMBC spectrum of the $[Am(PTEH)_3](OTf)_3$ complex in CD_3OD.

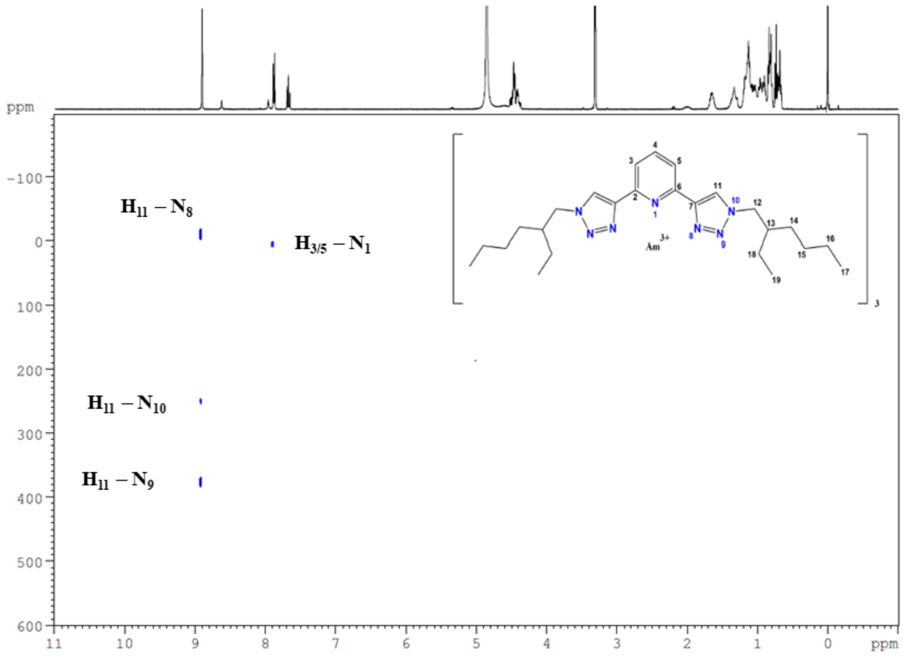

Figure S31: $^{1}H,^{15}N$ HMQC spectrum of the $[Am(PTEH)_3](OTf)_3$ complex in CD_3OD.

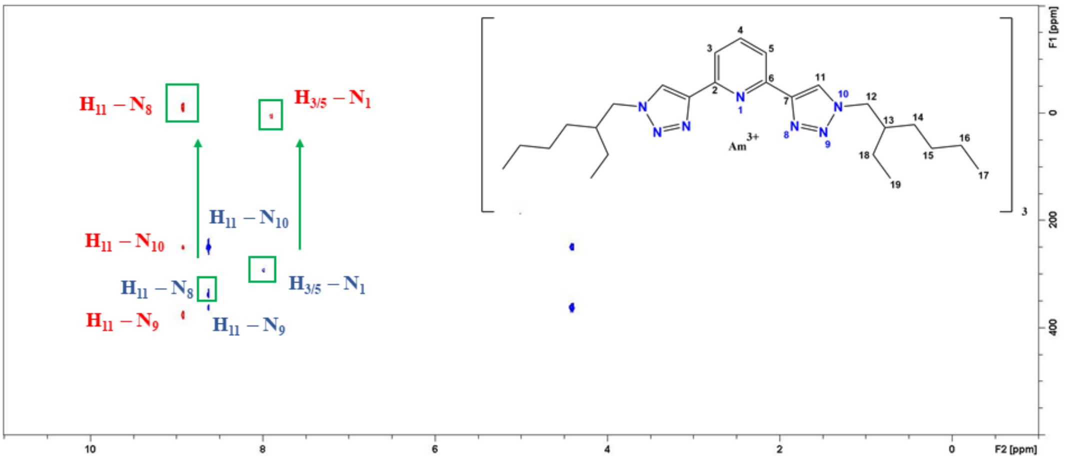

Figure S32: Overlay of two ^{1}H, ^{15}N-HMQC spectra of $[Am(PTEH)_3](OTf)_3$ complex and free ligand. Correlations of the Am(III) complex are in red, whereas those of the free ligand are in blue.

Temperature-dependent experiments

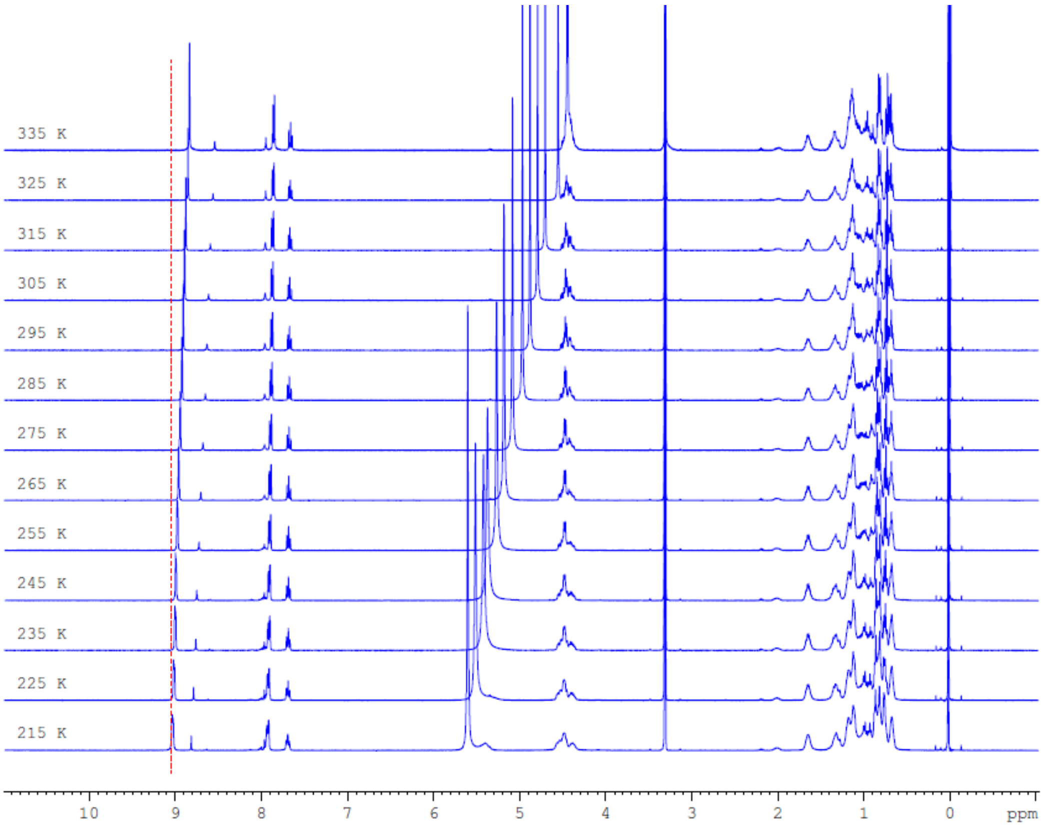

Figure S33: 1D ^{1}H spectra of $[Am(PTEH)_3](OTf)_3$ complex in CD_3OD at increasing temperature.

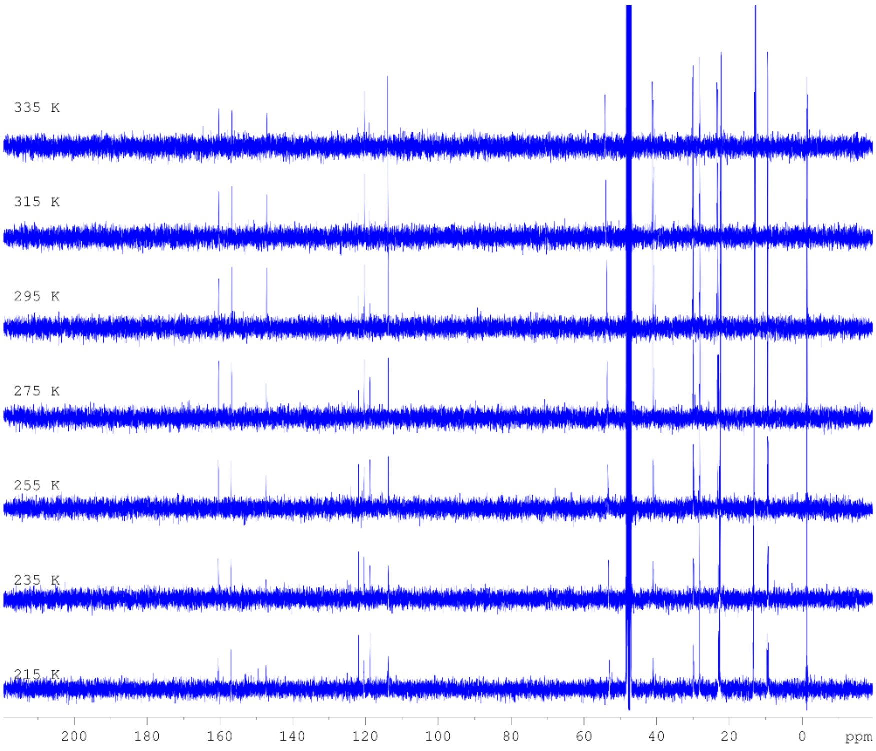

Figure S34: Stacked plot of 1D ^{13}C spectra of $[Am(PTEH)_3](OTf)_3$ complex in CD_3OD at increasing temperature.

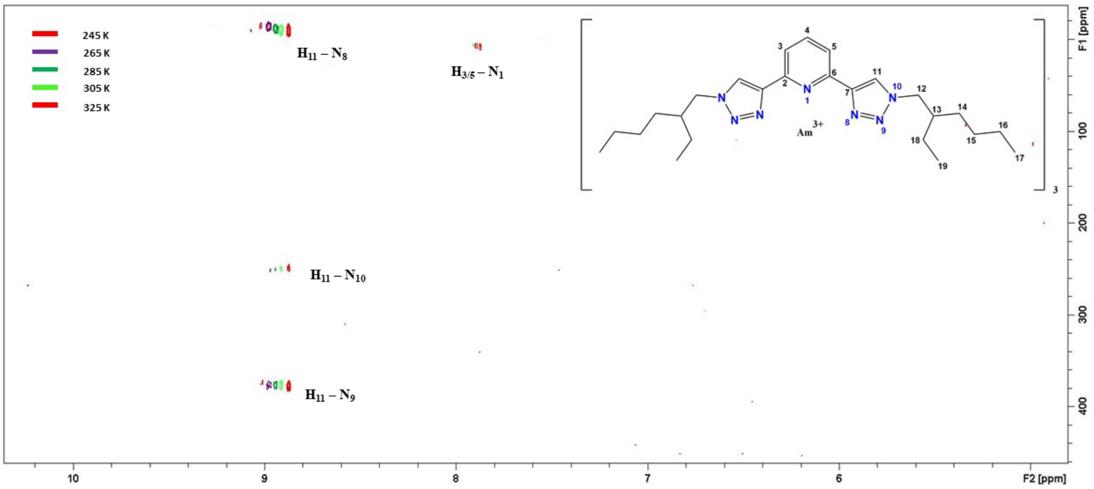

Figure S35: ^{1}H, ^{15}N-HMQC spectrum of $[Am(PTEH)_3](OTf)_3$ complex in CD_3OD at increasing temperature.

Further phenomena were found in the 1:3 $[Am(PTEH)_3](OTf)_3$ complex by comparing the 1D ^{1}H and ^{13}C spectra with those of the 1:3 $[Lu(PTEH)_3](OTf)_3$ complex. All the protons belonging to the pyridine and the triazole moieties in the 1:3 $[Am(PTEH)_3](OTf)_3$ complex appear shifted upfield compared to the equivalent signals in the 1:3 $[Lu(PTEH)_3](OTf)_3$ complex (Figure S36). In the 1D ^{13}C spectra of the 1:3 $[Am(PTEH)_3](OTf)_3$ spectrum, all carbon atoms close to the coordinating nitrogen atoms and the farthest carbon of the pyridine moiety appear shifted downfield whereas the remaining carbon signals appear shifted upfield with respect to the 1:3 $[Lu(PTEH)_3](OTf)_3$ complex. This could be summarized as positive chemical shift difference ($\delta_{Am} > \delta_{Lu}$) and negative chemical shift difference ($\delta_{Am} < \delta_{Lu}$). Protons of the pyridine and the triazole show a positive chemical shift difference whereas carbon atoms show alternating positive and negative chemical shift differences along the ligand aromatic core (Figure S37). All these qualitative observations could be attributed to a different bonding nature in actinides and lanthanides complexes.

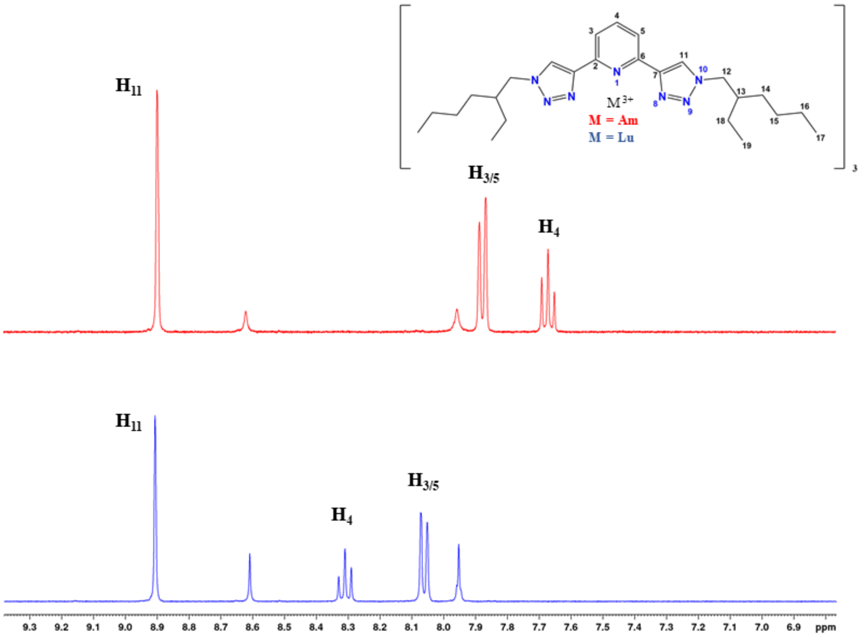

Figure S36: Aromatic region of the stacked plot ^{1}H NMR spectra for $[Am(PTEH)_3](OTf)_3$ (*top*) and $[Lu(PTEH)_3](OTf)_3$ (*bottom*) complexes.

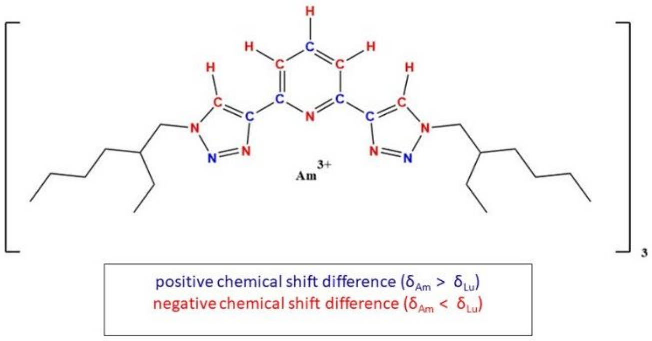

Figure S37: Aromatic chemical shift differences between the $[Am(PTEH)_3](OTf)_3$ and the $[Lu(PTEH)_3](OTf)_3$ complexes.

REFERENCES

[1] Harrison, A., Energy-resolved mass spectrometry: a comparison of quadrupole cell and cone-voltage collision-induced dissociation, *Rapid communications in mass spectrometry* 13.16 (1999), pp. 1663–1670.

[2] Miranda J Keith-Roach. "A review of recent trends in electrospray ionisation–mass spectrometry for the analysis of metal–organic ligand complexes". In: Analytica chimica acta 678.2 (2010), pp. 140–148.

[3] Sonia Colette et al. “Use of electrospray mass spectrometry (ESI-MS) for the study of europium (III) complexation with bis (dialkyltriazinyl) pyridines and its implications in the design of new extracting agents”. In: *Inorganic chemistry* 41.26 (2002), pp. 7031–7041.
